# Ion‐Electron Fusion Transparent Film for Interactive Soft Robotics

**DOI:** 10.1002/advs.202516816

**Published:** 2025-10-13

**Authors:** Zhiqiu Ye, Chao Zhang, Gaoyang Pang, Kaichen Xu, Shuangjia Liu, Yichen Wang, Jiahuan Qiu, Huaixuan Dai, Bingru Wang, Yinliang Gan, Liu Yang, Huayong Yang, Geng Yang

**Affiliations:** ^1^ State Key Laboratory of Fluid Power and Mechatronic Systems School of Mechanical Engineering Zhejiang University Hangzhou 310000 P. R. China; ^2^ School of Electrical and Computer Engineering The University of Sydney Sydney 2006 Australia; ^3^ MOE Key Laboratory of Macromolecular Synthesis and Functionalization Department of Polymer Science and Engineering Zhejiang University Hangzhou 310000 P. R. China; ^4^ Polytechnic Institute Zhejiang University Hangzhou 310000 P. R. China; ^5^ Department of Energy Environmental and Chemical Engineering Washington University St. Louis MI 63 130 USA; ^6^ ZJU‐UIUC Institute Zhejiang University Haining 314400 P. R. China; ^7^ College of Optical Science and Engineering Zhejiang University Hangzhou 310000 P. R. China; ^8^ State Key Laboratory of Fluid Power and Mechatronic Systems the School of Mechanical Engineering the Zhejiang Engineering Research Center of Robotics in Electric Equipment Manufacturing and Intelligent Operation‐Maintenance and the Zhejiang Key Laboratory of Intelligent Operation and Maintenance Robot Zhejiang University Hangzhou 310000 P. R. China

**Keywords:** bioinspired underwater robots, interactive soft robotics, strain sensors, stretchable transparent electrodes, visible light communication

## Abstract

Marine organisms combine sensory networks and bioluminescence to achieve adaptive interaction in complex environments. Existing bioinspired soft robots, however, mainly focus on actuation and rarely integrate both functionalities to enhance autonomy. Flexible transparent conductive films offer a promising route for strain‐based proprioception and visible light communication, but maintaining stable conductivity and high transparency under large deformations remains challenging. Here, An ion‐electron fusion film is presented, termed i‐PEDOT:PSS, comprising a poly(3,4‐ethylenedioxythiophene):poly(styrene‐sulfonate) (PEDOT:PSS) layer on an ionic substrate. The interfacial ion penetration, in combination with the pre‐formed microcrack structure, imparts i‐PEDOT:PSS with robust conductivity and desirable strain‐sensing capability under large strain. The i‐PEDOT:PSS achieves a highly linear and repeatable electromechanical response over strains up to 300%, approximately three times that of previously reported transparent strain sensors, while maintaining an optical transmittance of 93%. Benefiting from these properties, i‐PEDOT:PSS serves as a multifunctional component in soft robotic systems. As a strain sensor, it enables real‐time monitoring and adjustment of the locomotion state of underwater transparent soft robots. As a stretchable transparent electrode, it supports electroluminescent devices for underwater optical signal transmission. This work established a fully soft, interactive robotic platform, offering a new framework for the development of perceptive and communicative soft robotics.

## Graphical abstract

An ion‐electron fusion transparent film with stable conductivity, high optical transmittance, and a broad linear strain‐sensing range of up to 300% is developed, attributed to the synergistic effects of ion penetration and microcrack design. When applied to soft robots, this film imparts proprioceptive sensing and underwater visible light communication capabilities, paving the way for fully soft interactive robotic systems.

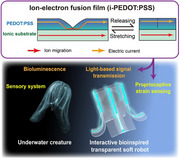

## 1 Introduction

Marine organisms in nature utilize sensory networks and bioluminescence for effective interaction within complex environments.^[^ ^1^, ^2^, ^3^, ^4^ ^]^ Sensory networks provide proprioception and exteroception, enabling adaptive closed‐loop responses critical for survival in hostile habitats.^[^ ^5^ ^]^ Bioluminescence, exemplified by jellyfish and Dosidicus gigas, allows reliable visual communication to broadcast state and intent to nearby conspecifics, particularly in dark conditions.^[^ ^1^, ^6^, ^7^ ^]^ By mimicking these features, soft robots gain state awareness and optical messaging capabilities, enhancing autonomy and coordination, especially in low‐visibility environments.^[^ ^8^, ^9^, ^10^ ^]^ Integrating these sensing and communication capabilities into soft robotic systems hinges on flexible, transparent, and conductive materials. These materials can serve as stretchable electrodes in deformable light‐emitting devices, facilitating visible light communication with advantages such as high bandwidth, low latency, and secure data transmission, which are particularly useful in dark environments.^[^ ^11^, ^12^ ^]^ Additionally, their optical transparency enables effective camouflage while functioning simultaneously as flexible sensors to detect environmental changes or mechanical deformation.^[^ ^13^ ^]^ Thus, achieving stable conductivity and high‐performance sensing properties under significant mechanical deformation, while maintaining high optical transmittance, is crucial for effective integration of flexible transparent conductive materials into interactive soft robotic systems.^[^ ^14^, ^15^ ^]^

Recently, a wide range of stretchable transparent conductive films has been developed based on various classes of conductive materials, such as conductive nanomaterials, conductive polymers, and ionic conductors. As to films based on conductive nanomaterials such as Ag nanowires (AgNWs) and carbon nanotubes (CNT), 2D or 3D structural designs are typically employed to achieve compliance under mechanical deformation.^[^ ^16^, ^17^ ^]^ However, the mechanical mismatch between the intrinsically rigid nanomaterials and the stretchable polymer substrate can lead to cracks or interfacial delaminations under strain, which results in extremely high resistance and potential electrical disconnections.^[^ ^18^, ^19^ ^]^ Furthermore, increasing the concentration of conductive nanomaterials to achieve high conductivity often comes at the expense of optical transmittance. Conductive polymers, such as poly(3,4‐ethylenedioxythiophene):poly(styrene‐sulfonate) (PEDOT:PSS), represent another widely used class of transparent conductors due to their intrinsic transparency, high electrical conductivity, facile doping tunability, and facile solution processability.^[^ ^20^ ^]^ Various techniques, including laser‐based strategies, have enabled PEDOT:PSS to be patterned onto flexible substrates, facilitating the rapid fabrication of customized functional devices capable of stable, long‐term signal recording and stimulation.^[^ ^21^, ^22^, ^23^ ^]^ Nevertheless, PEDOT:PSS lacks the mechanical compliance required for integration into highly deformable soft robots.^[^ ^24^ ^]^ In contrast, intrinsically soft ionic conductors, such as hydrogels and ionic gels, offer excellent stretchability and high optical transparency, rendering them promising candidates for interactive soft robotic systems.^[^ ^25^ ^]^ Various processing strategies, including freeze‐casting and salting‐out techniques, have been developed to improve the mechanical robustness.^[^ ^26^, ^27^, ^28^ ^]^ Nonetheless, their relatively low electrical conductivity limits their application scenarios such as stretchable electrodes for light‐emitting devices.^[^ ^29^ ^]^ Additionally, hydrogel‐based conductors are prone to dehydration, which leads to poor long‐term stability, particularly under elevated temperatures.^[^ ^30^ ^]^ Therefore, achieving stable conductivity under large mechanical deformation while maintaining high optical transmittance remains a critical and ongoing challenge in the development of stretchable transparent conductive films.

**Figure 1:**
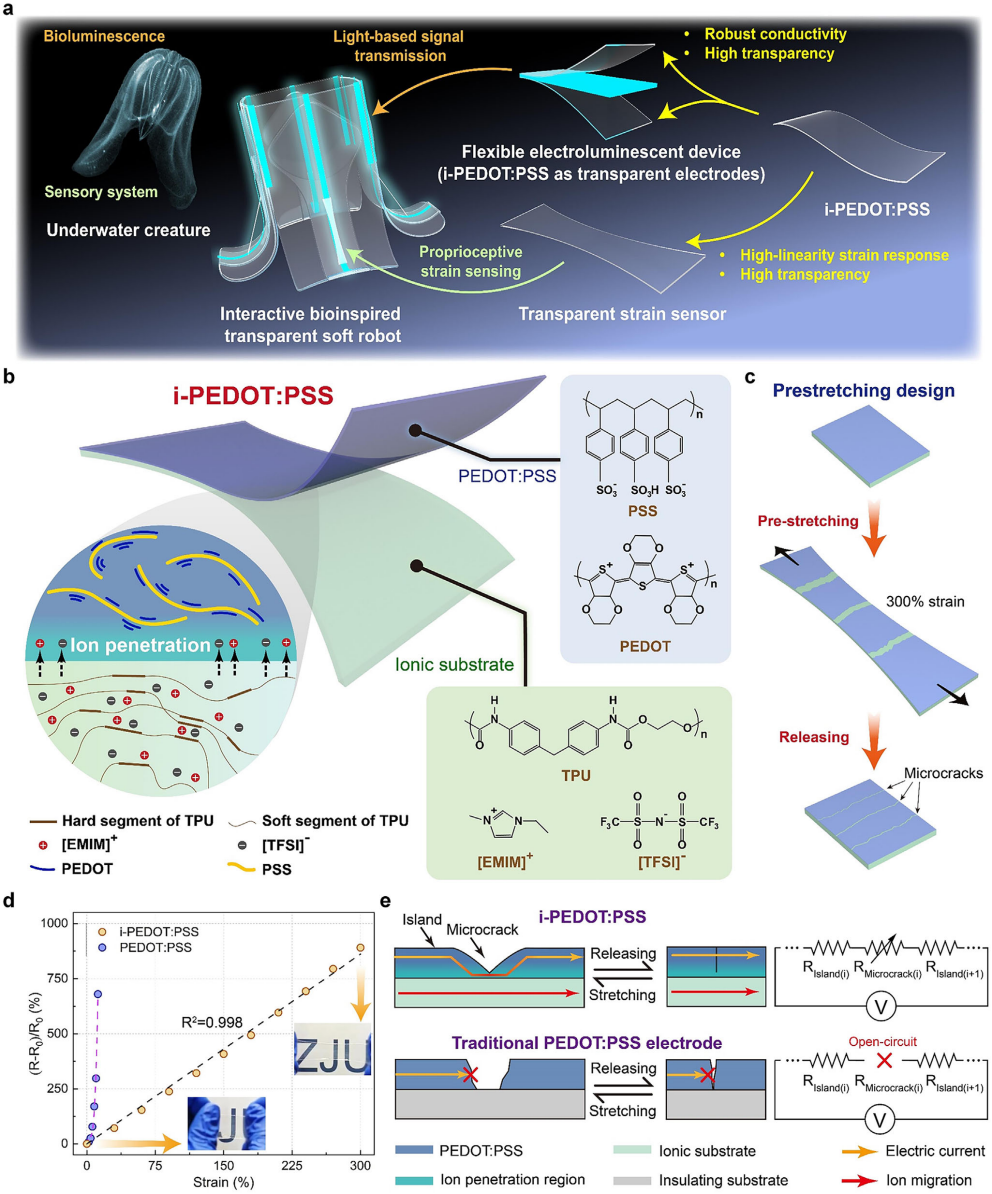
Prototyping of i‐PEDOT:PSS film. a) Application of i‐PEDOT:PSS in interactive transparent soft robotic system. b) Schematic illustration of the i‐PEDOT:PSS architecture. c) Pre‐stretching‐induced microcrack formation. d) Comparison between i‐PEDOT:PSS film and pristine PEDOT:PSS on insulating substrate. e) The conduction and strain‐sensing mechanism of i‐PEDOT:PSS film during stretching.

In this paper, we develop an ion‐electron fusion stretchable transparent film (i‐PEDOT:PSS), comprising an ionic gel substrate and a PEDOT:PSS layer. Ion penetration at the bilayer interface enables the combination of the high conductivity of PEDOT:PSS and the high stretchability of ionic gel. A pre‐stretching process is further employed to induce microcracks within the PEDOT:PSS layer, endowing the i‐PEDOT:PSS with strain sensing capability. The developed i‐PEDOT:PSS exhibits three key properties: i) the interfacial ion penetration enables stable conductivity under dynamic, large deformation (up to 300% strain), significantly surpassing that of pristine PEDOT:PSS (≈13% strain); ii) coupled ion penetration and microcrack propagation yield a highly linear, repeatable electromechanical response over a strain range of up to 300%, which is approximately three times the range of existing transparent strain sensors; iii) the inherent optical transparency of both the ionic gel substrate and PEDOT:PSS delivers an overall transmittance of 93%. These combined properties render the i‐PEDOT:PSS film a promising candidate for interactive soft robotic systems (**Figure** **1**a). In particular, the broad linear strain‐sensing range effectively minimizes the impact of potential pre‐strain on sensing accuracy, which can arise during integration with soft robotic systems. As a result, i‐PEDOT:PSS can serve as an effective strain sensor for underwater transparent soft robots, enabling real‐time posture monitoring and closed‐loop feedback control. Furthermore, the high transparency and stable conductivity under large deformation make it well‐suited for use as an electrode layer in stretchable alternating current electroluminescent (ACEL) devices. When incorporated into soft robotic platforms, the i‐PEDOT:PSS‐based ACEL devices facilitate optical signal encoding and transmission, enabling underwater temperature monitoring and positional tracking. The unprecedented fully soft perceptive and communicative robotic system proposed in this work provides a new framework for future research.

## 2 Results

### 2.1 Prototyping of i‐PEDOT:PSS Film

Figure 1b presents a schematic illustration of the i‐PEDOT:PSS architecture, which features a bilayer configuration consisting of an ionic substrate and a PEDOT:PSS layer. Thermoplastic polyurethane (TPU) functions as the elastic matrix of the ionic substrate, while the incorporated ionic liquid, 1‐ethyl‐3‐methylimidazolium bis(trifluoromethylsulfonyl)imide ([EMIM][TFSI]), imparts ionic conductivity to the substrate. Ions in the substrate can migrate toward the PEDOT:PSS layer, leading to the formation of an interfacial ion penetration region.^[^ ^31^ ^]^ This ion‐enriched interface establishes an effective coupling between the layers, synergistically combining the high electrical conductivity of PEDOT:PSS with the superior stretchability of the ionic substrate. The characterization and validation of ion penetration will be detailed in the following section. To ensure stable and repeatable electrical performance under large tensile strains, the i‐PEDOT:PSS film is pre‐stretched to 300% strain to induce microcracks within the PEDOT:PSS layer (Figure 1c). These microcracks can subsequently open and close during cyclic stretching and releasing, effectively absorbing the tensile stress within the film and preventing additional structural damage. Owing to the synergy effect of ion penetration and microcrack design, i‐PEDOT:PSS demonstrates superior conductive stability and repeatability over a broad strain range (0%–300%), as well as a highly linear strain‐sensing response. This performance significantly outperforms that of conventional PEDOT:PSS‐based conductive films fabricated on insulating substrates (TPU), which are prone to irreversible mechanical failure under large tensile deformation (Figure 1d). To ensure a valid comparison, the thickness of the PEDOT:PSS layer in both cases is controlled to ≈0.42 µm, achieved by spin‐coating at 1000 rpm for 4 s on the ionic substrate and at 850 rpm for 4 s on the TPU substrate after a 120 s plasma treatment. Detailed fabrication process of i‐PEDOT:PSS is illustrated in Figure S1 (Supporting Information). Notably, owing to the addition of fluorosurfactant in the PEDOT:PSS solution prior to film formation, the wetting property at the ionic substrate–PEDOT:PSS interface is enhanced, resulting in an interfacial adhesion strength of ≈400 N m^−1^ and ensuring a stable connection between the two layers (Figure S2, Supporting Information).

The conduction and strain‐sensing mechanism of i‐PEDOT:PSS during stretching is outlined as follows (Figure 1e). The ionic liquid, serving as a plasticizer, softens the PSS‐rich domains and attenuates the electrostatic interactions between PEDOT and PSS chains, thereby introducing soft phases into the rigid semi‐crystalline PEDOT:PSS matrix. This structural modulation moderately enhances the conductivity of the PEDOT:PSS layer by promoting PEDOT aggregation and crystallinity, thus facilitating the formation of continuous conductive pathways and improving charge transport (Table S1, Supporting Information). It also increases the mechanical adaptability of the ion‐penetration region, allowing it to accommodate substantial deformation.^[^ ^32^ ^]^ As a result, partially intact ion penetrated‐PEDOT:PSS at the cracked section continue to support electronic conduction. Simultaneously, the underlying ionic substrate, owing to its intrinsic stretchability, remains unbroken throughout deformation, maintaining a stable ionic conductive pathway.^[^ ^33^ ^]^ The progressive widening of microcracks reduces the proportion of intact PEDOT:PSS regions, resulting in a gradual decline in electronic conductivity and an increased reliance on ionic conductivity. This transition leads to a corresponding increase in the total impedance of the i‐PEDOT:PSS film. Upon release of the applied tensile strain, the elasticity of the ionic substrate facilitates the gradual contact of the opposing fracture surfaces in the PEDOT:PSS layer. With the progressive increase in the contact area, conductive pathways are re‐established, leading to a reduction in resistance (Figure S3, Supporting Information).

The electrical conduction behavior of the i‐PEDOT:PSS film can be modeled as a series configuration of resistive elements associated with the cracked regions (microcracks) and uncracked regions (islands). Owing to the negligible variation in electrical conductivity within the uncracked regions during mechanical deformation, these segments can be reasonably approximated as constant resistors (Figure S4, Supporting Information). In contrast, the cracked regions exhibit progressive and uniform crack propagation with increasing tensile strain, resulting in a nearly linear decrease in overall conductivity. The broad linear strain‐sensing range of the i‐PEDOT:PSS film provides an effective strategy to decouple unintended pre‐strain during integration with soft robots, highlighting its strong potential as a reliable strain sensor for interactive soft robotic systems. The microscopy of crack widening and reclosure is depicted in Figure S5 (Supporting Information). In contrast, traditional PEDOT:PSS films deposited on insulating substrates lack both the plasticizing effect provided by ionic liquids and the ion penetration layer necessary to maintain electrical interconnection under mechanical deformation. As a result, these films exhibit poor stretchability and are prone to irreversible fracture when subjected to tensile strain. Once a crack occurs, their conductive pathways cannot be restored even after the strain is released, rendering them unsuitable for applications requiring large and reversible mechanical deformation.

### 2.2 Validation of Ion Penetration Process

**Figure 2:**
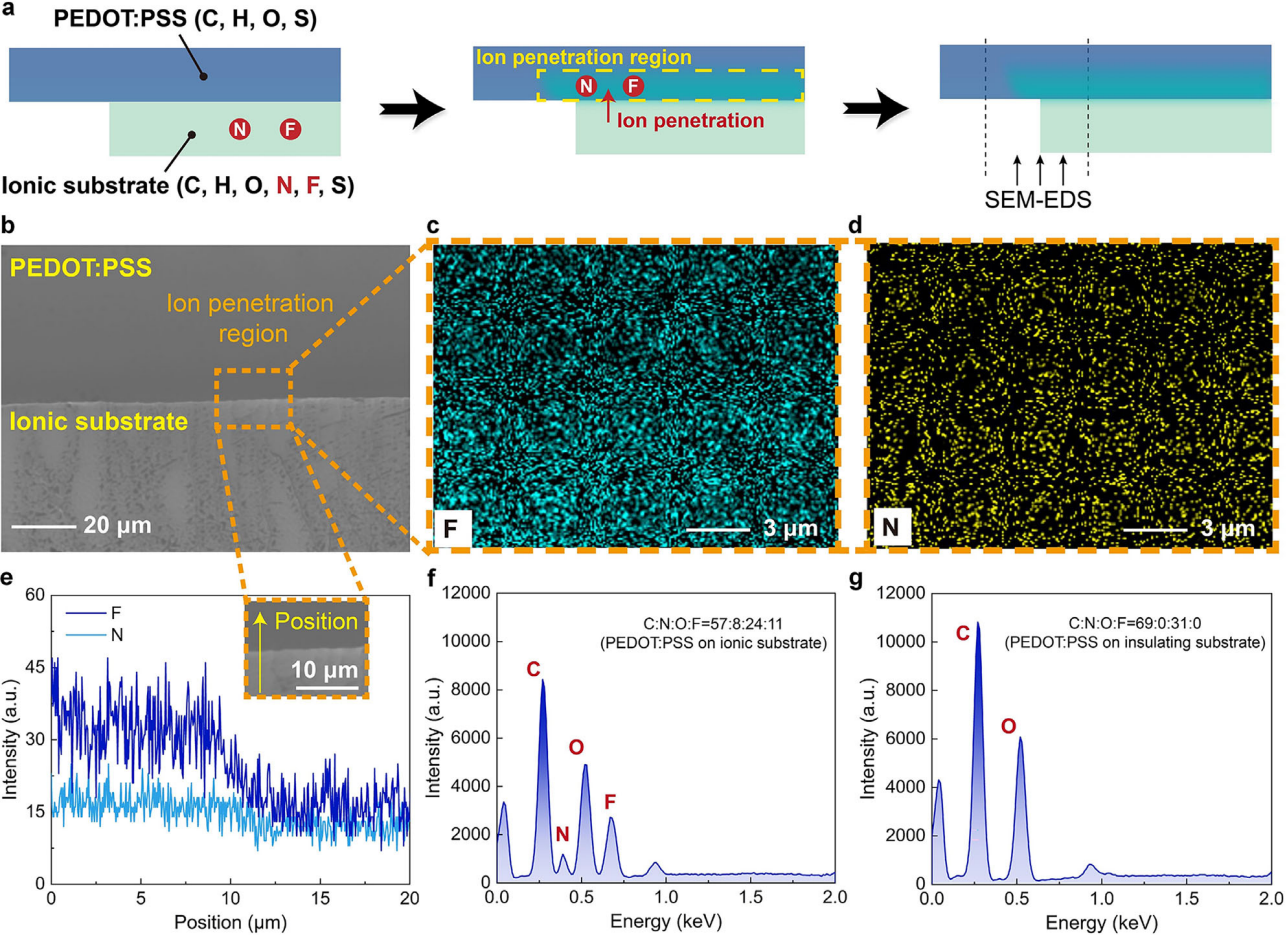
Validation of the ion penetration of i‐PEDOT:PSS. a) Schematic illustration of the experimental setup. b) SEM image. Scale bar: 20 µm. c) SEM‐EDS mapping of element F. Scale bar: 3 µm. d) SEM‐EDS mapping of element N. Scale bar: 3 µm. e) Line‐scanning profile across the yellow arrow shown in the inset. Scale bar: 10 µm. f) SEM‐EDS spectrum of PEDOT:PSS on the ionic substrate. g) SEM‐EDS spectrum of PEDOT:PSS on insulating substrate.

The stable conductivity of the i‐PEDOT:PSS during stretching is primarily attributed to ion penetration at the interface between the PEDOT:PSS layer and the ionic substrate. Based on the molecular formula shown in Figure 1b, the primary elements in the ionic substrate include Carbon (C), Hydrogen (H), Oxygen (O), Nitrogen (N), Fluorine (F), and Sulfur (S), whereas PEDOT:PSS contains only C, H, O, and S. To investigate the ion penetration process, a PEDOT:PSS‐ionic substrate bilayer structure is characterized using scanning electron microscopy (SEM) in conjunction with corresponding energy‐dispersive X‐ray spectroscopy (EDS) elemental mapping techniques (**Figure** **2**a).

Figure 2b demonstrates the SEM image of the examined region, while the EDS elemental mapping images of F and N on the surface are presented in Figure 2c,d. The presence of F and N signals within the PEDOT:PSS layer near the interface strongly suggests ion penetration from the ionic substrate. The line‐scanning profile further confirms the ion penetration process, as evidenced by a slight decrease in F and N content at the boundary (Figure 2e). Moreover, the SEM‐EDS spectrum of PEDOT:PSS on the ionic substrate is depicted in Figure 2f. The atomic ratio of C/N/O/F is evaluated to be 57/8/24/11, indicating the presence of F and N. In contrast, no signals of F and N are detected in the SEM‐EDS spectrum of a pure PEDOT:PSS film (Figure 2g). Collectively, these results provide compelling evidence of ion penetration from the ionic substrate into the PEDOT:PSS layer.

### 2.3 Characterization of i‐PEDOT:PSS Film

**Figure 3:**
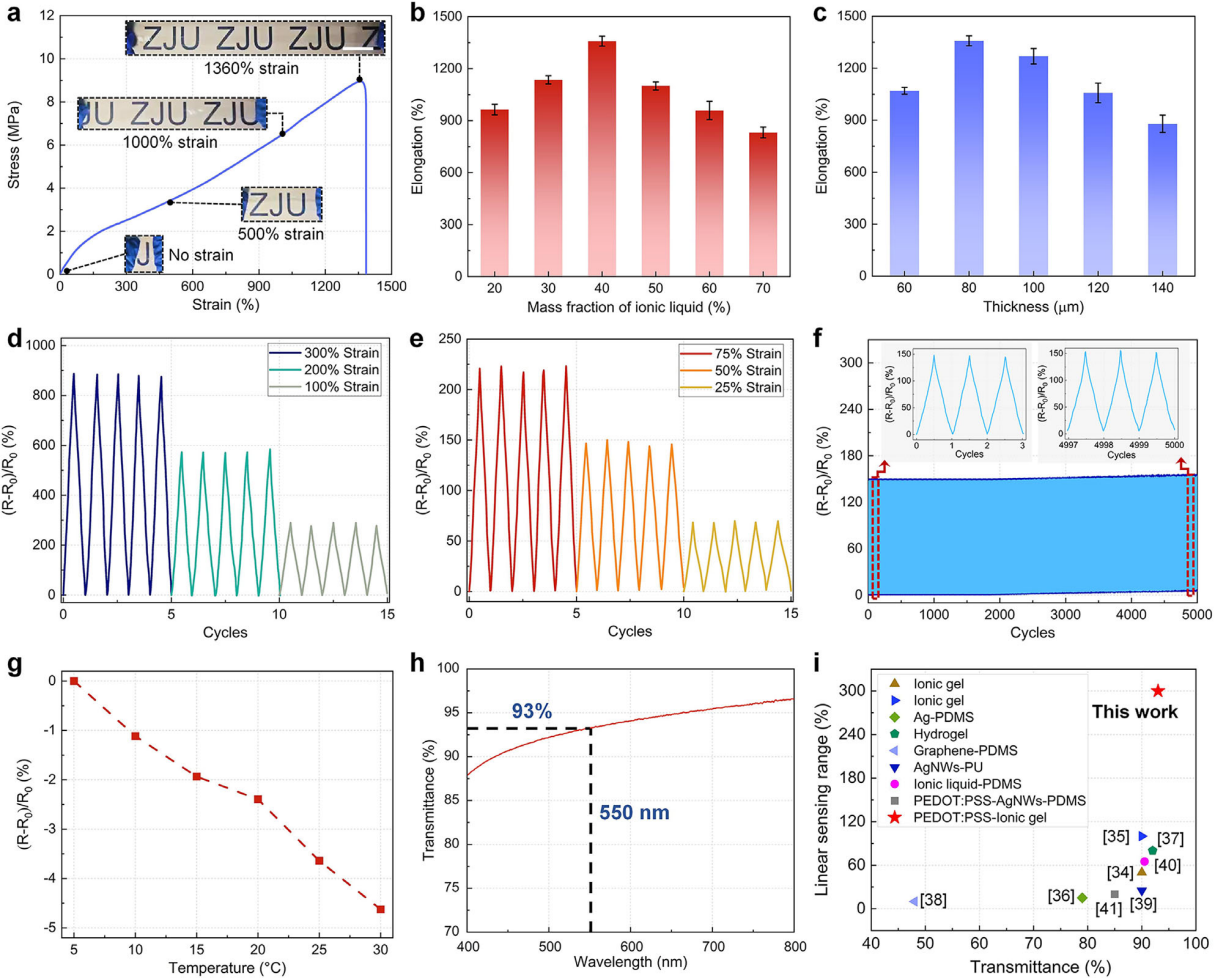
Mechanical, electrical, and optical characterization of i‐PEDOT:PSS film. a) The stress–strain curve of the ionic substrate. Scale bar: 3 cm. b) Correlation between the mechanical property of ionic substrate and ionic liquid fraction. Data represent the mean ± s.d. (*n*  =  3 independent samples). c) Correlation between the mechanical property of ionic substrate and its thickness. Data represent the mean ± s.d. (*n*  =  3 independent samples). d) Dynamic resistance tests under the strain of 100%, 200%, and 300%. e) Dynamic resistance tests under the strain of 25%, 50%, and 75%. f) Durability test of i‐PEDOT:PSS (50% strain). g) Resistance‐temperature curve of i‐PEDOT:PSS. h) Optical transmittance spectra of i‐PEDOT:PSS. i) Comparison with previously reported stretchable transparent films for strain‐sensing application.^[^ ^34^, ^35^, ^36^, ^37^, ^38^, ^39^, ^40^, ^41^ ^]^

To evaluate the mechanical performance of i‐PEDOT:PSS, we examine the stress–strain behavior of the ionic substrate, which primarily determines the stretchability of the i‐PEDOT:PSS composite. This approach effectively eliminates the fabrication‐related variations in PEDOT:PSS layer across different samples, thereby enhancing the reliability of the assessment. The stress–strain curve of the ionic substrate reveals exceptional stretchability, with fracture occurring at a strain of ≈1360% (**Figure** **3**a; Movie S1, Supporting Information). The dimensions and size parameters of the samples are designed in accordance with ISO 37:2017 (Figure S6, Supporting Information). As shown in Figure 3b,c, the mechanical properties of the ionic substrate depend on both its thickness and the ionic liquid fraction, with detailed data presented in Figure S7 (Supporting Information). It should be noted that although the ionic liquid exhibits a plasticizing effect, excessive content reduces the stretchability of the ionic substrate by diluting the polymer network, weakening inter‐chain interactions, and ultimately compromising the overall mechanical properties. Dynamic mechanical testing of the ionic substrate with optimized parameters (80 µm thickness and 40% ionic liquid content) further confirms excellent elastic recovery under cyclic strain between 0% and 300% (Figure S8, Supporting Information). However, when the tensile strain is increased to 350%, the film exhibits residual plastic deformation after complete unloading. Therefore, the operational strain range for i‐PEDOT:PSS is set to be 0%–300%, which serves as the basis for the pre‐stretching parameter in Figure 2b.

The resistance response of i‐PEDOT:PSS under uniaxial stretching is shown in Figure 1d, demonstrating a broad linear strain‐sensing range from 0% to 300% with a gauge factor of 2.87. Meanwhile, Figure S9 (Supporting Information) demonstrates that multiple samples exhibit similar crack morphology and density, and all samples maintain high linearity with gauge factor variations below 9%, indicating that the sensing performance is consistent and closely associated with microcrack formation. To further investigate its electrical performance under dynamic loading, dynamic resistance tests are conducted at various strain levels. As depicted in Figure 3d,e, the resistance response remains highly stable across different strain amplitudes. In contrast, the pure ionic gel maintains high repeatability under dynamic stretching up to 300% strain—attributable to its mechanical robustness, but suffers from limited linearity and a low gauge factor ranging from 0.09 to 0.33 (Figure S10, Supporting Information). These shortcomings constrain its applicability in interactive underwater soft robots, which underscores the advantage of integrating the ionic gel with PEDOT:PSS to achieve both high stretchability and enhanced sensing performance. Furthermore, a durability test of 5000 stretching cycles at 50% strain is carried out (Figure 3f). The results indicate only a minor drift of ≈7% in resistance, confirming the robustness and reliability of i‐PEDOT:PSS under repeated dynamic strain. At an extreme strain of 300%, the peak resistance increases by ≈15% after 1000 dynamic stretching cycles, which is attributed to the formation of minor microcracks during repeated deformation (Figure S11, Supporting Information). Notably, after accounting for the shift in baseline resistance at zero strain, the relative resistance change is limited to only 7.5% over 1000 cycles. This indicates that baseline compensation can effectively minimize measurement deviation even under 300% strain, further confirming the robust strain‐sensing performance of i‐PEDOT:PSS. In addition, the transient response of i‐PEDOT:PSS is characterized. As shown in Figure S12 (Supporting Information), the response time under a transient strain of 50% is only 60 ms, highlighting the great potential of this material for dynamic strain sensing in underwater robotic applications.

The proposed i‐PEDOT:PSS is intended for underwater applications, where water temperature fluctuates between 5 ° and 30 °C. Therefore, it is essential to evaluate the resistance stability of i‐PEDOT:PSS across this temperature range. As shown in Figure 3g, the resistance variation of i‐PEDOT:PSS remains below 5% within the tested range, indicating a low temperature sensitivity. This characteristic is advantageous for ensuring the performance reliability of i‐PEDOT:PSS when employed as a transparent electrode or strain sensor in underwater soft robots.

Optical transmittance is another critical parameter for stretchable transparent conductive films. The high transparency makes i‐PEDOT:PSS well‐suited for specific applications, such as strain sensors for transparent soft robots with camouflage demands, or as electrode layers in flexible optoelectronic devices for visible‐light communication among robots. The effect of the spin‐coating parameters of i‐PEDOT:PSS on the optical and electrical performance is provided in Figure S13, Table S2 (Supporting Information), with the corresponding thickness of the PEDOT:PSS layer depicted in Figure S14 (Supporting Information). Taking both the transmittance and electrical conductivity of i‐PEDOT:PSS under different processing parameters into account, the optimal spin‐coating parameter for PEDOT:PSS is determined to be 1000 rpm for 4 s. At this setting, the i‐PEDOT:PSS film exhibits an electrical conductivity of 31.25 S cm^−1^, which is significantly higher than that of the pure ionic gel (0.0001 S cm^−1^). Meanwhile, i‐PEDOT:PSS maintains a transmittance above 87% across the visible range (400–760 nm), reaching 93% at the characteristic wavelength of 550 nm (Figure 3h).

Furthermore, a comparative analysis with previously reported stretchable transparent films for strain‐sensing application is presented in Figure 3i; Table S3 (Supporting Information).^[^ ^34^, ^35^, ^36^, ^37^, ^38^, ^39^, ^40^, ^41^ ^]^ The comparison result highlights the superior linear strain‐sensing range and transparency of i‐PEDOT:PSS, underscoring its potential as a promising candidate for next‐generation interactive soft robotic systems.

### 2.4 The i‐PEDOT:PSS‐Based Strain Sensing for Soft Robotic Locomotion Adjustment

Sensors serve as essential functional components in interactive soft robotic systems, enabling both proprioception and exteroception, thereby bridging the gap toward real‐world applications involving interactions with both the environment and humans. Among these sensory functions, accurate perception of a robot's posture is crucial for ensuring that it follows its intended locomotion. However, in soft robotics, the integration of flexible sensors presents unique challenges due to the mutual deformability of the sensors and the robot body. This often results in the pre‐strain of the sensors during installation, thereby requiring post‐installation recalibration—an effort‐intensive and time‐consuming process. If the sensor exhibits high linearity across its full measurement range—meaning its sensitivity remains nearly constant under deformation—such recalibration can be avoided. Moreover, high linearity markedly reduces the computational complexity involved in strain calculation.

**Figure 4:**
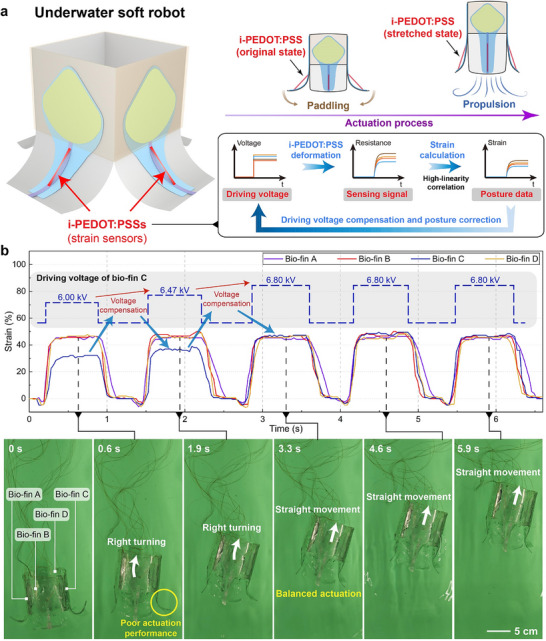
Strain sensing and locomotion adjustment for underwater transparent soft robot. a) Operation principle of i‐PEDOT:PSS in interactive soft robotic systems. b) Locomotion demonstration of the strain sensor‐integrated underwater robot. Scale bar: 5 cm.

In this work, i‐PEDOT:PSS, with a broad linear strain‐sensing range, serves as a stretchable transparent strain sensor and is integrated into an underwater transparent soft robot for real‐time posture detection and correction, as illustrated in **Figure** **4**a. The technical details of the soft robot are provided in our previous work.^[^ ^42^ ^]^ The robot comprises four electro‐hydraulic actuators, namely bio‐fins A, B, C, and D. When a high‐voltage is applied, positive and negative charges accumulate on the outer surfaces of the actuators, generating electrostatic Maxwell stress that drives the liquid dielectric toward the tail of the actuator. Assisted by a pre‐buckling bracket that constrains the actuator in a predefined bending posture at rest state, the rapid influx of liquid dielectric provides sufficient bearing force for the actuator to straighten swiftly. The resulting interaction with the surrounding water produces a reaction force that propels the actuator forward (Figure S15, Supporting Information). This deformation simultaneously imposes uniaxial tensile strain on the i‐PEDOT:PSS strain sensors, both ends of which are affixed to actuators. The strain signals directly reflect the actuation performance of the electro‐hydraulic actuators. By comparing the outputs from the four i‐PEDOT:PSS sensors, the overall actuation state of the underwater soft robot can be comprehensively evaluated. This enables timely identification of potential trajectory deviations caused by performance degradation in one or more actuators, allowing prompt implementation of correction strategies.

Figure 4b; Movie S2 (Supporting Information) illustrate the underwater locomotion of the soft robot integrated with i‐PEDOT:PSS sensors. Despite being intended for linear motion, the robot deviates rightward as a result of diminished performance in bio‐fin C, which is promptly detected by the i‐PEDOT:PSS strain sensors. Based on the strain‐sensing signals from the four channels, the negative feedback control strategy is then implemented by increasing the driving voltage of bio‐fin C from 6 to 6.8 kV. This adjustment balances the performance of the four actuators and achieved linear motion of the underwater soft robot. The multichannel strain signal acquisition circuit and detailed negative feedback control strategy are depicted in Figures S16 and S17 (Supporting Information).

In contrast, underwater soft robots without integrated strain sensors cannot obtain real‐time posture information, which may lead to trajectory deviations due to performance degradation in one or more actuators (Figure S18, Supporting Information). Notably, the optical transparency of the i‐PEDOT:PSS preserves the inherent passive camouflage capability of the transparent soft robot in the underwater environment, further underscoring its application potential for interactive soft robotic systems.

### 2.5 ACEL Devices Based on i‐PEDOT:PSS for Interactive Soft Robotic Systems

Bioluminescence serves as a crucial means of communication among biological organisms, offering valuable inspiration for the development of interactive robotic systems. This concept is particularly pertinent to underwater robots since such low‐light conditions are inherently favorable for the propagation of optical signals, making visible light communication a highly effective strategy for underwater applications. Given that water exhibits relatively high transparency to blue and green light (450–550 nm), flexible ACEL devices, which typically emit in the blue‐green spectrum, are particularly well‐suited for underwater robotic systems.^[^ ^43^ ^]^ The luminescence mechanism of flexible ACEL devices is primarily based on the electronic excitation and radiative recombination of phosphor particles under a high‐frequency, high‐voltage alternating electric field. When the voltage is applied, the periodic electric field excites electrons in the emissive layer. These electrons are captured by luminescent centers and, upon returning to the valence band, emit photons as visible light at specific wavelengths.

**Figure 5:**
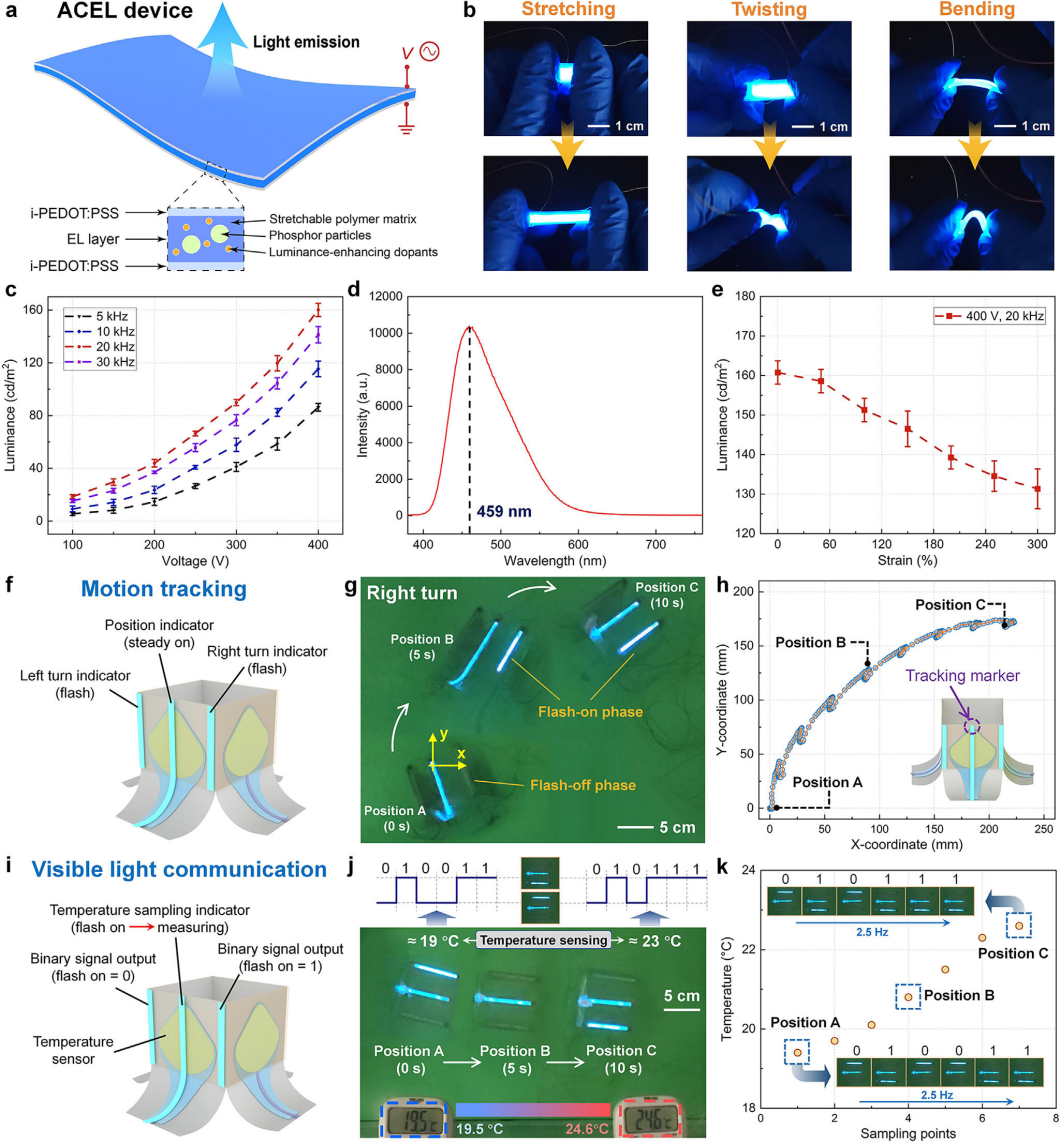
ACEL devices based on i‐PEDOT:PSS for interactive soft robotic systems. a) Structure and principle of i‐PEDOT:PSS based ACEL devices. b) Luminance performance under stretching, twisting, and bending deformations. Scale bar: 1 cm. c) Correlations between luminance of ACEL devices and voltage parameters (amplitude and frequency). Data represent the mean ± s.d. (*n* = 3 independent samples). d) Electroluminescent spectrum of the device. e) Correlations between luminance and strain at a voltage of 400 V and frequency of 20 kHz. Data represent the mean ± s.d. (*n* =  3 independent samples). f) Schematic illustration and g) Demonstration of interactive underwater soft robots for motion tracking. Scale bar: 5 cm. h) The real‐time captured trajectory of the robot via image‐based recognition. i) Schematic illustration and j) Demonstration of interactive underwater soft robots for temperature sensing and optical signal transmission. Scale bar: 5 cm. k) The measured temperature values and the corresponding optical binary signals along the robot's trajectory.

In this study, we develop flexible ACEL devices based on i‐PEDOT:PSS electrodes (**Figure** **5**a). The electroluminescent (EL) layer consists of a stretchable polymer matrix, phosphor particles, and luminance‐enhancing dopants.^[^ ^44^ ^]^ The robust and stable conductivity of i‐PEDOT:PSS under significant mechanical deformation ensures that the ACEL devices maintain consistent high luminescence performance under stretching, bending, and twisting conditions (Figure 5b; Figure S19, Movie S3, Supporting Information). In contrast, electrodes composed of pristine PEDOT:PSS on insulating substrates exhibit insufficient stretchability, leading to irreversible damage and device failure even under minor deformation. Meanwhile, ACEL devices based solely on ionic gels also suffer from poor luminance performance. The conductivity of ionic gel electrodes relies on ion migration. When a voltage is applied, conductive ions accumulate near the electrode lead regions, leading to a non‐uniform ion distribution. Upon stretching, the device elongates, further aggravating the uneven ion distribution and weakening the electric field strength in the central region, which becomes insufficient to excite the phosphors embedded in the EL layer. As a result, light emission is predominantly localized near the electrode leads. Upon release of strain and recovery of the device to its original length, the ion distribution becomes more uniform, restoring the central electric field and enabling uniform light emission across the entire device. Notably, the overall luminescence intensity of the ionic gel‐based ACEL devices remains lower than i‐PEDOT:PSS‐based ACEL devices due to the inherently lower conductivity of pure ionic gels. These findings demonstrate that the proposed i‐PEDOT:PSS film, combining the high conductivity of PEDOT:PSS with the excellent stretchability of ionic gels, is an ideal stretchable transparent electrode material for flexible ACEL devices.

Experiments on the luminance performance of i‐PEDOT:PSS‐based ACEL devices are further conducted. The luminance of the ACEL devices increases monotonically with the voltage amplitude, with an optimized voltage frequency of 20 kHz (Figure 5c). The mass ratio between the luminance‐enhancing dopants and the phosphor particles also has a positive effect on the luminance (Figure S20, Supporting Information). In this study, a ratio of 0.4 is selected, as further increasing the dopant content could raise the viscosity of the mixture and potentially introduce structural defects into the device during fabrication. The electroluminescent spectrum of the device, shown in Figure 5d, is centered at 459 nm, with a full width at half‐maximum of 85 nm. The ACEL devices maintain stable luminance under strain ranging from 0% to 300%. A slight decrease in luminance is observed, caused by the minor reduction in the conductivity of i‐PEDOT:PSS under stretching (Figure 5e). A durability test is further conducted on the ACEL device under a strain of 50%. The luminance is measured when the device returns to its original length. The results indicate only a minor luminance drift of ≈3%, confirming the robustness and reliability of the i‐PEDOT:PSS‐based ACEL device under repeated dynamic strain (Figure S21, Supporting Information).

We further integrated the i‐PEDOT:PSS‐based ACEL devices into an underwater soft robot to enable motion tracking and optical signal transmission in dark environments. Three ACEL devices are mounted on the robot: a longer middle device, operated at 250 V for continuous illumination to facilitate real‐time localization, and two shorter side ACEL devices, operated at 400 V for higher luminance and used for signaling left or right turns via flashing (Figure 5f,g). The voltage supply circuit of the ACEL device is designed to enable feasible adjustment of the luminance state of the device (Figure S22, Supporting Information). By designating the top of the middle ACEL device as a tracking marker, the robot's position and trajectory could be dynamically captured (Figure 5h; Movie S4, Supporting Information).

Additionally, the ACEL‐integrated underwater soft robot demonstrated capabilities for visible light communication of sensory signals (Figure 5i; Table S4, Supporting Information). A temperature gradient is established within a water tank by positioning a heater and a cooler at opposite ends (Figure S23, Supporting Information), resulting in measured temperatures of 19.5 ° and 24.6 °C at the starting and ending points of robot's motion trajectory, respectively. Considering the temperature insensitivity of i‐PEDOT:PSS (Figure 3g), the fan‐shaped pure ionic gel in the robot actuator is applied to enable underwater temperature sensing. The conductivity of the pure ionic gel arises from the migration of cations and anions, which is strongly temperature‐dependent. Elevated temperatures promote ion‐pair dissociation, generating more free ions, and simultaneously enhance ionic mobility through thermal activation. This combined effect significantly enhances the ionic gel's conductivity at elevated temperatures, endowing it with high‐temperature sensitivity. According to the experimental results in Figure S24 (Supporting Information), an ionic gel with a 20% ionic liquid fraction exhibits the highest sensitivity, which is selected for temperature sensing. Since the fan‐shaped ionic gel also serves as the electrode for the high‐voltage electro‐hydraulic actuator, we conducted experiments in which its resistance was continuously monitored over 1000 actuation cycles (Figure S25, Supporting Information). The results show no obvious trend in resistance variation, with the overall change remaining within 2.5%, indicating that the effect of heat generation on the ionic gel during actuation is negligible.

During locomotion, the robot performs temperature measurements after each propulsion using the resistance–temperature curve of the ionic gel at 15°–30 °C (Figure S26, Supporting Information), as indicated by the illumination of the central ACEL device. The two side ACEL devices encode temperature information at the start and end positions using binary signals—the illumination of the respective device representing binary “1” or “0” (Figure 5j; Movie S5, Supporting Information). The measured temperature values and the corresponding optical binary signals along the robot's trajectory are summarized in Figure 5k, with a measurement error of less than 10% compared to the standard thermometer. The fully soft interactive robotic system integrated with sensing and communication functions demonstrates the promising potential for applications in underwater exploration and cooperative multi‐robot operations.

## 3 Conclusion

In this study, we develop an ion‐electron bilayer fusion transparent film (i‐PEDOT:PSS) by depositing PEDOT:PSS onto an ionic substrate. The resulting i‐PEDOT:PSS demonstrates significantly enhanced stretchability compared to pristine PEDOT:PSS on an insulating substrate, which is attributed to the ion penetration at the interface between the PEDOT:PSS layer and the ionic substrate. Microcracks within the PEDOT:PSS layer are further induced through a pre‐stretching process. Leveraging the synergistic effects of ion penetration and microcrack formation, i‐PEDOT:PSS achieves a broad linear strain‐sensing range of 0–300%, significantly surpassing previously reported transparent strain sensors. Additionally, i‐PEDOT:PSS exhibits a high optical transmittance of 93%. Take advantage of the aforementioned properties, i‐PEDOT:PSS is employed as a strain sensor for underwater transparent soft robots, enabling real‐time monitoring and adjustment of the locomotion state of underwater transparent soft robots. It also serves as a transparent electrode in ACEL devices, which are integrated into the soft robot for optical signal encoding and transmission, enabling underwater temperature monitoring and positional tracking. The application of i‐PEDOT:PSS to underwater soft robots provides a novel template for future research on interactive soft robotic systems.

Looking ahead, the circuits developed in this study could be further miniaturized and implemented in flexible form. In the current design, the actuators, ACEL devices, and strain sensors are powered and controlled via separate external modules connected by wires. While this setup allows straightforward operation, it constrains the robot's workspace and adaptability. As the number of devices increases, the wiring complexity increases, potentially interfering with the robot's motion and overall performance. Integrating functional chips onto flexible, transparent substrates to form flexible circuits could minimize the impact on the robot's flexibility and transparency, thereby preserving its performance in practical underwater applications.^[^ ^45^ ^]^

## 4 Experimental Section

### Preparation of the i‐PEDOT:PSS

The i‐PEDOT:PSS was composed of an ionic gel substrate and a PEDOT:PSS layer. The fabrication process involves three steps: PEDOT:PSS solution pre‐treatment, ionic substrate fabrication, and i‐PEDOT:PSS formation (Figure S1, Supporting Information).

In the first step, the PEDOT:PSS solution was filtered and dispensed onto the surface of ethylene glycol (EG). After maintaining the solution at 20 °C for 2 h, the upper layer of the PEDOT:PSS‐EG mixture was extracted. This process promotes phase separation between PEDOT and PSS, removing excess PSS and enhancing the conductivity of the PEDOT:PSS.^[^ ^46^ ^]^ To improve the wetting properties, 1 wt.% Zonyl‐FS300 fluorosurfactant was added, facilitating the deposition of the PEDOT:PSS solution onto the ionic substrate in the third step.^[^ ^47^ ^]^

In the second step, TPU granules (Shore 60A) and dimethyl formamide (DMF) are blended in a 1:6 (w/w) ratio and stirred at 80 °C for 12 h using a magnetic stirrer (HSC‐19T, Joanlab Equipment Co., Ltd.) at 1000 rpm. Next, ionic liquid ([EMIM][TFSI]) was added to the solution and stirred at 80 °C for another 12 h to ensure uniform mixing. The mixture was then degassed in a vacuum chamber for 10 min to eliminate bubbles and prevent surface defects in the ionic substrate. A glass slide was cleaned ultrasonically in deionized water, ethanol, and acetone for 2 min each. The ionic mixture was applied to the glass slide and evenly spread using a spin coater (KW‐4A, Chemat Technology Inc.) at 800 rpm for 4 s. Finally, the ionic gel was cured at 80 °C for 12 h to fully remove any residual DMF solvent.

In the third step, the pre‐treated PEDOT:PSS solution was dispensed onto the ionic gel substrate and evenly spread using the spin coater (1000 rpm, 4 s). The sample was cured at 100 °C for 1 h to remove residual solvent and form the i‐PEDOT:PSS film. The film was peeled off from the glass and pre‐stretched to 300% strain, then released to induce microcracks within the PEDOT:PSS layer.

For applications in underwater soft robotics, the i‐PEDOT:PSS film was further encapsulated with silicone rubber (Ecoflex 00‐31, Smooth‐On, Inc.) to achieve waterproofing.

### Preparation of ACEL Devices Based on Different Electrodes

The EL layer was composed of a stretchable polymer matrix, phosphor particles, and luminance‐enhancing dopants. Ecoflex 00‐31 was selected as the polymer matrix due to its excellent stretchability and high optical transparency. ZnS:Cu (Shanghai Keyan Phosphor Technology Co., Ltd.) and BaTiO_3_ particles (500 nm) are employed as the phosphor particles and luminance‐enhancing dopants, respectively. The entire fabrication process of the EL layer was depicted in Figure S27 (Supporting Information). The polymer matrix, phosphor particles, and dopants are mixed at a mass ratio of 1:1:0.4. The mixture was stirred at 500 rpm for 2 min, then degassed in a vacuum chamber for 5 min to remove bubbles and minimize surface defects in the EL layer. The resulting mixture was blade‐coated onto a glass slide pretreated with a release agent (Ease Release 200, Smooth‐On, Inc.), with the thickness controlled at 50 µm. The EL layer was then cured at 100 °C for 10 min and subsequently peeled off. Plasma treatment on both sides of the EL layer using an oxygen plasma cleaner (TS‐PL02, Tonosn Tech Co., Ltd) was performed to enhance wettability, enabling the deposition of the three types of electrodes described in this work onto its surfaces and forming a sandwich‐structured ACEL device.

### Characterization

A universal test machine (34SC‐05, Instron) was used to stretch i‐PEDOT:PSS and evaluate its mechanical and electrical properties, while an DMM meter (34461A, Keysight) records the resistance of i‐PEDOT:PSS during stretching. The conductivity of the i‐PEDOT:PSS film was measured using a four‐probe resistance tester (HPS2662, Helpass). The optical transmittance of i‐PEDOT:PSS was measured with a UV–vis spectrophotometer (Cary 60, Agilent Technologies Inc.). The thickness of the PEDOT:PSS layer prepared under different spin‐coating parameters was measured using a surface profilometer (DEKTAK‐XT, Bruker). A high‐voltage amplifier (ATA‐2161 Aigtek) provides the AC voltage supply for the ACEL devices during luminance testing, and a luminance meter (LS‐100, Konica) measures their luminance. The electroluminescent spectrum of the ACEL devices was measured using a high‐sensitivity spectrometer (QE PRO, Ocean Optics).

### Circuit for Multichannel Strain Signal Acquisition

A multichannel strain signal acquisition circuit was designed to achieve real‐time measurement of i‐PEDOT:PSS strain sensors on four electro‐hydraulic actuators. The circuit consists of a microcontroller, an analog switch, an operational amplifier, and two multiplexer modules. The circuit schematic and chip selection are shown in Figure S16 (Supporting Information). A time‐division multiplexing strategy was adopted to sequentially activate each strain‐sensing unit, enabling multichannel strain signal acquisition. The microcontroller controls the vertical and horizontal multiplexers to connect the selected sensing unit to the operational amplifier. Based on the voltage signal at the circuit output, the resistance value can be derived. To avoid signal crosstalk caused by branch currents in unselected channels, an analog switch module was introduced. By grounding the unselected channels through controlled switching, effective electrical isolation was achieved, thereby enhancing the accuracy of signal acquisition.

### Circuit for the Voltage Supply of ACEL Devices

A high‐frequency voltage supply circuit was designed to dynamically control the voltage supply (amplitude, frequency, and on/off status) of the ACEL device in the interactive soft robot systems. This circuit consists of a boost converter, transformer, gate driver, and microcontroller. The circuit schematic and chip selection are shown in Figure S22 (Supporting Information). The two‐stage voltage boosting process begins with the boost converter, which steps up the 5 V DC input to a programmable DC voltage ranging from 5 to 15 V. The transformer then further amplifies the voltage with a fixed gain of 50. The gate driver ensures efficient switching and control of the transformer, allowing the microcontroller to regulate the transformer's output frequency. Thus, both the amplitude and frequency of the output voltage are tunable through microcontroller programming.

### Motion Tracking of the Underwater Soft Robot

The robot's trajectory was tracked using the TrackerMIL algorithm implemented in OpenCV. The top of the central ACEL device was designated as the tracking marker, enclosed within a bounding box in the initial frame. This bounding box was continuously updated throughout the entire locomotion process of robot. By recording the center coordinates of the bounding box in each frame, the complete motion path of the robot was accurately reconstructed.

## Conflict of Interest

The authors declare no conflict of interest.

## Supporting information



Supporting Information

Supplemental Movie 1

Supplemental Movie 2

Supplemental Movie 3

Supplemental Movie 4

Supplemental Movie 5

## Data Availability

The data that support the findings of this study are available from the corresponding author upon reasonable request.
