# Supplementary material for: Ion‐Electron Fusion Transparent Film for Interactive Soft Robotics
Source: Adv Sci (Weinh). 2025 Oct 13;13(8):e16816. doi: 10.1002/advs.202516816 (PMC12884758; doi:10.1002/advs.202516816)
Supplement: Supplementary file 1 — Supporting Information [file ADVS-13-e16816-s005.docx]

Supporting Information for

**Ion-electron fusion transparent film for interactive soft robotics**

Zhiqiu Ye, Chao Zhang*, Gaoyang Pang, Kaichen Xu, Shuangjia Liu, Yichen Wang, Jiahuan Qiu, Huaixuan Dai, Bingru Wang, Yinliang Gan, Liu Yang, Huayong Yang, Geng Yang*

*Corresponding author: Chao Zhang, Geng Yang

Email: chao.zhang@zju.edu.cn; yanggeng@zju.edu.cn

**This PDF file includes:**

**Figure S1.** Fabrication process of i-PEDOT:PSS.

**Figure S2**. 90° Peeling test of the i-PEDOT:PSS film.

**Figure S3**. Schematic illustration of the electrical contact process during dynamic stretching.

**Figure S4.** Resistance change of i-PEDOT:PSS under tensile loading during and after the pre-stretching process.

**Figure S5.** SEM images of microcrack widening and reclosure in i-PEDOT:PSS.

**Figure S6.** Dimension and shape of tested samples of ionic substrate.

**Figure S7.** Stress–strain curves of ionic substrates with varying thicknesses and ionic liquid fractions.

**Figure S8.** Mechanical response of ionic substrates under dynamic tensile strain.

**Figure S9.** Surface morphology and sensing performance of different batches.

**Figure S10.** Electrical characterization of ionic gel.

**Figure S11.** Durability test of i-PEDOT:PSS (300% strain).

**Figure S12.** Transient response of i-PEDOT:PSS under 50% strain.

**Figure S13.** Optical transmittance of i-PEDOT:PSS with different PEDOT:PSS spin-coating parameters.

**Figure S14.** Thickness of the PEDOT:PSS layer prepared under different spin-coating parameters.

**Figure S15.** Actuation mechanism of the electro-hydraulic actuators.

**Figure S16.** Multichannel strain signal acquisition circuit.

**Figure S17.** Negative feedback control for an underwater soft robot with i-PEDOT:PSS strain sensors.

**Figure S18.** Trajectory deviations of an underwater soft robot without integrated strain sensors.

**Figure S19.** Luminance performance of ACEL devices based on i-PEDOT:PSS, PEDOT:PSS, and ionic gel.

**Figure S20.** Correlations between luminance of ACEL devices and mass ratio in the EL layer.

**Figure S21.** Durability test of the ACEL devices under dynamic strain of 50%.

**Figure S22.** Circuit for voltage supply to ACEL devices.

**Figure S23.** Experimental setup for temperature sensing and visible light communication applications.

**Figure S24.** The resistance–temperature curves of ionic gels with different ionic liquid fractions.

**Figure S25.** Resistance variation of the ionic gel during continuous high-voltage actuation.

**Figure S26.** Resistance-temperature curve of ionic gel (20% ionic liquid fraction) at 15–30°C.

**Figure S27.** Fabrication process of the EL layer in ACEL devices.

**Table S1.** Comparison of PEDOT:PSS film on pure TPU and ionic substrate

**Table S2.** Properties of i-PEDOT:PSS with different PEDOT:PSS spin-coating parameters.

**Table S3.** Comparison with previously reported stretchable transparent films for strain-sensing application.

**Table S4.** Luminance states of ACEL devices in the application of visual light communication.

Legends for **Movie S1**. Stretchability of i-PEDOT:PSS film.

Legends for **Movie S2**. The i-PEDOT:PSS-based strain sensing for soft robotic posture correction.

Legends for **Movie S3**. Comparative luminance performance of ACEL devices employing various electrodes.

Legends for **Movie S4**. The i-PEDOT:PSS-based ACEL devices for robot motion tracking.

Legends for **Movie S5**. The i-PEDOT:PSS-based ACEL devices for visible light communication.

SI References

**Other supporting materials for this manuscript include the following:**

**Movies S1 to S5**





Figure S1. Fabrication process of i-PEDOT:PSS. a) PEDOT:PSS solution pre-treatment. b) Ionic substrate fabrication. c) i-PEDOT:PSS fabrication. EG is the abbreviation for ethylene glycol.


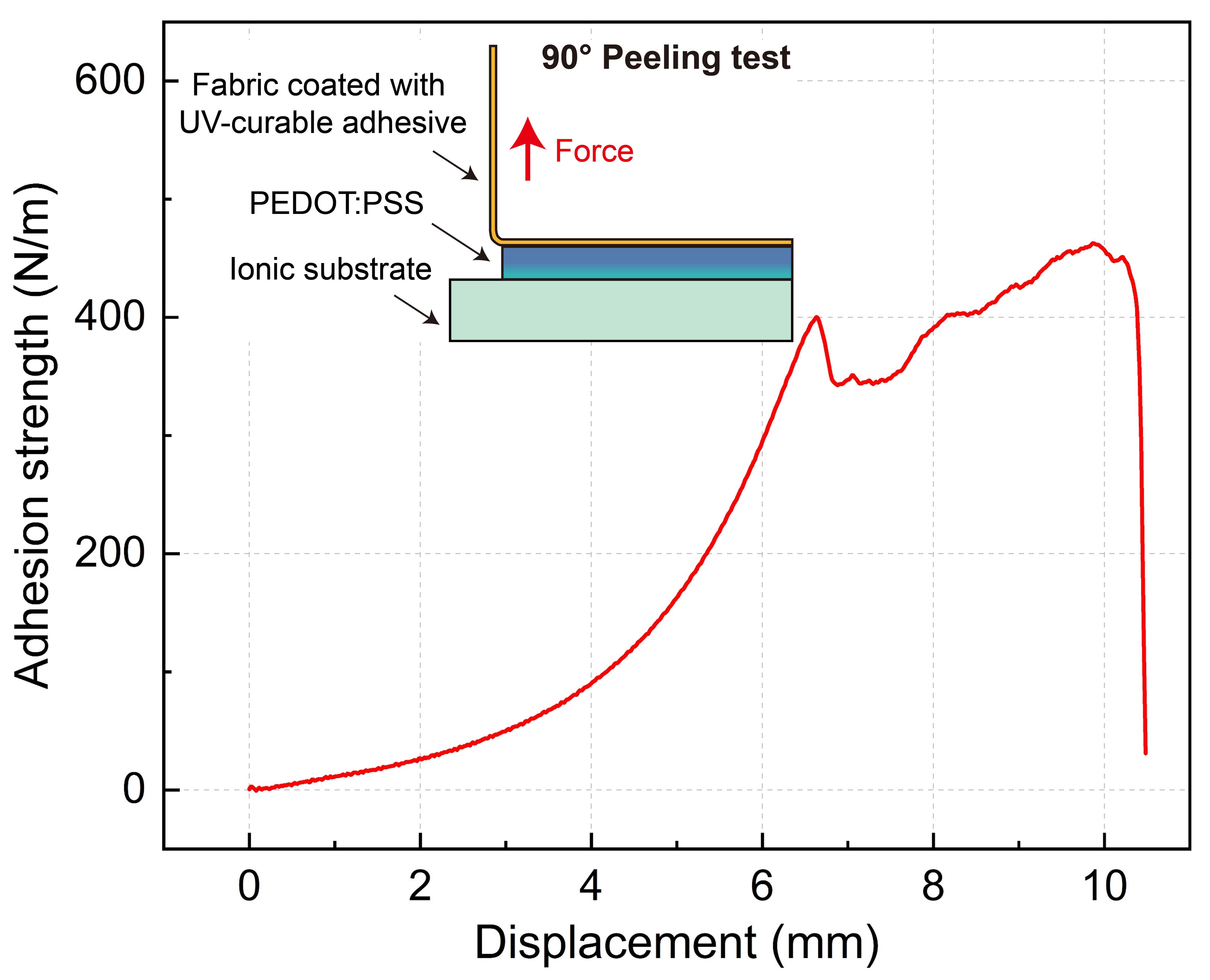


Figure S2. 90° Peeling test of the i-PEDOT:PSS film.


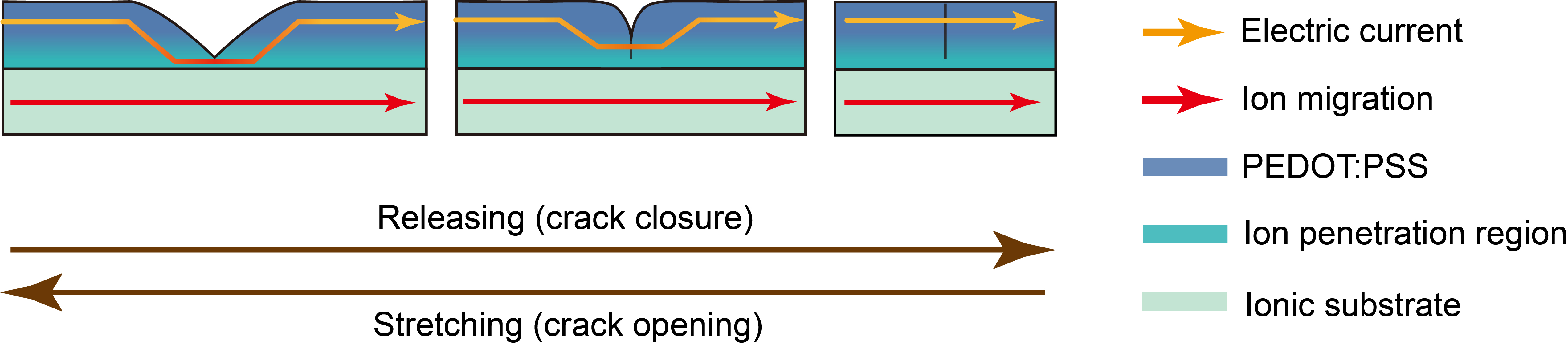


Figure S3. Schematic illustration of the electrical contact process during dynamic stretching.


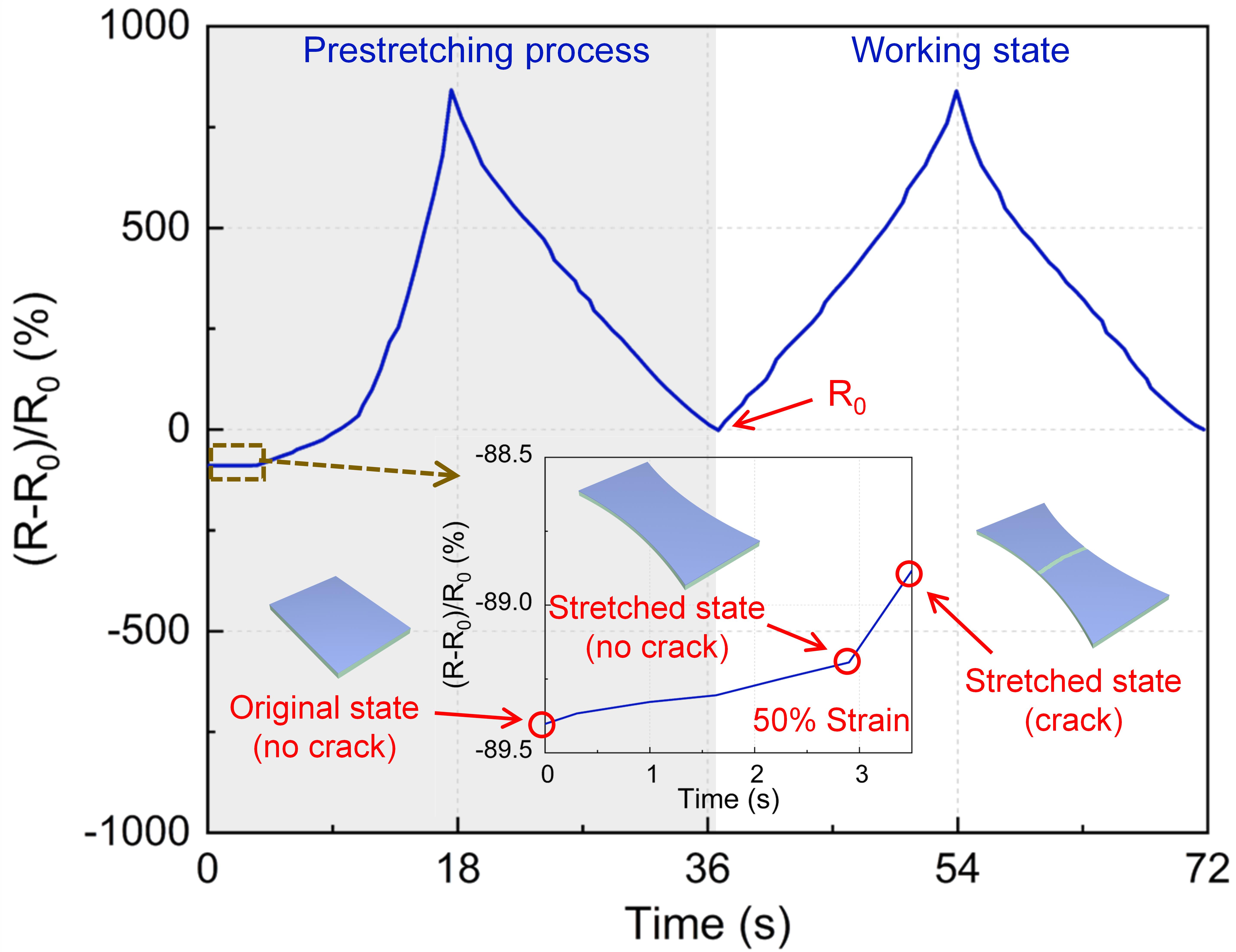


Figure S4. Resistance change of i-PEDOT:PSS under tensile loading during and after the pre-stretching process. The resistance remains nearly unchanged until cracks occur at strain of 50% within PEDOT:PSS layer, suggesting stable conductivity in the uncracked regions.


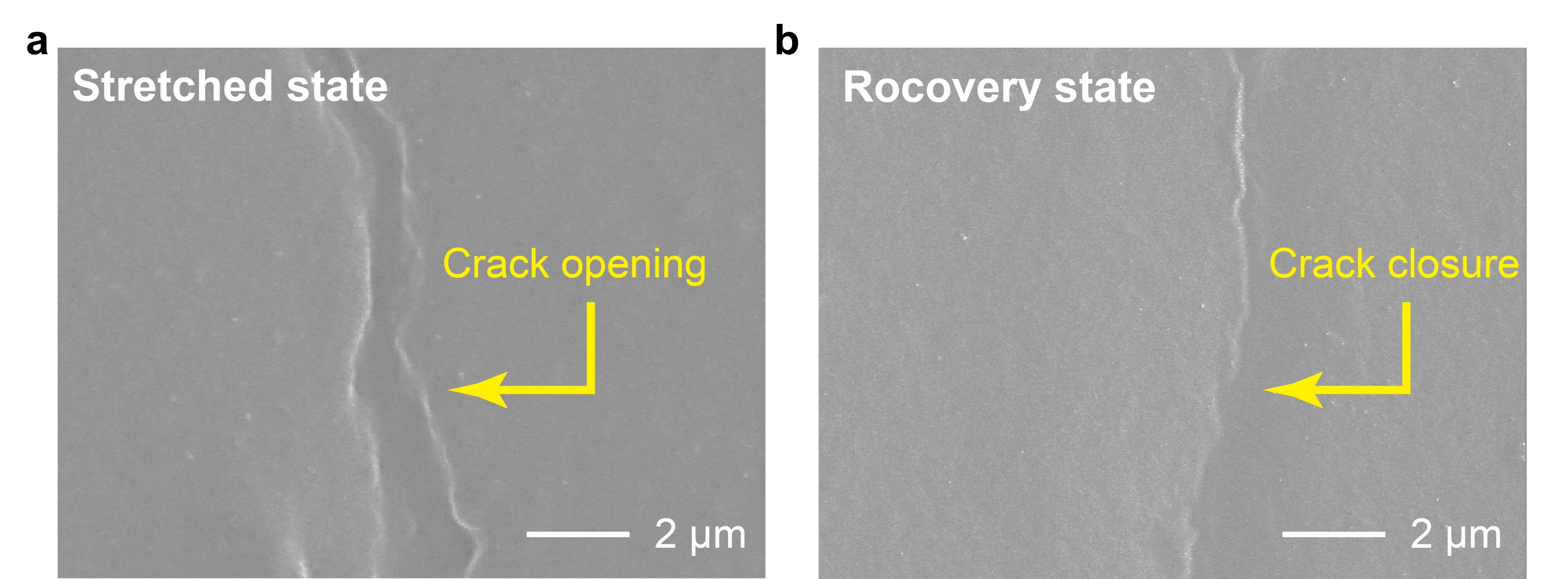


Figure S5. Scanning electron microscopy images of microcrack a) widening during stretching and b) reclosure during releasing in i-PEDOT:PSS. Scale bar: 2 µm.


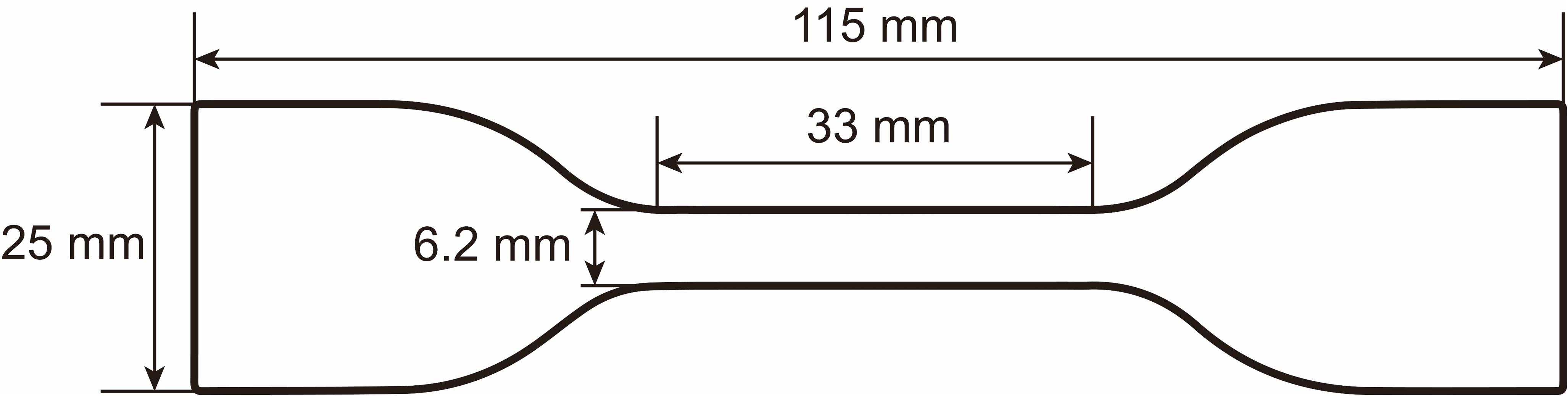


Figure S6. Dimension and shape of tested samples of ionic substrate.





Figure S7. Stress–strain curves of ionic substrates with varying a) ionic liquid fractions and b) thicknesses.


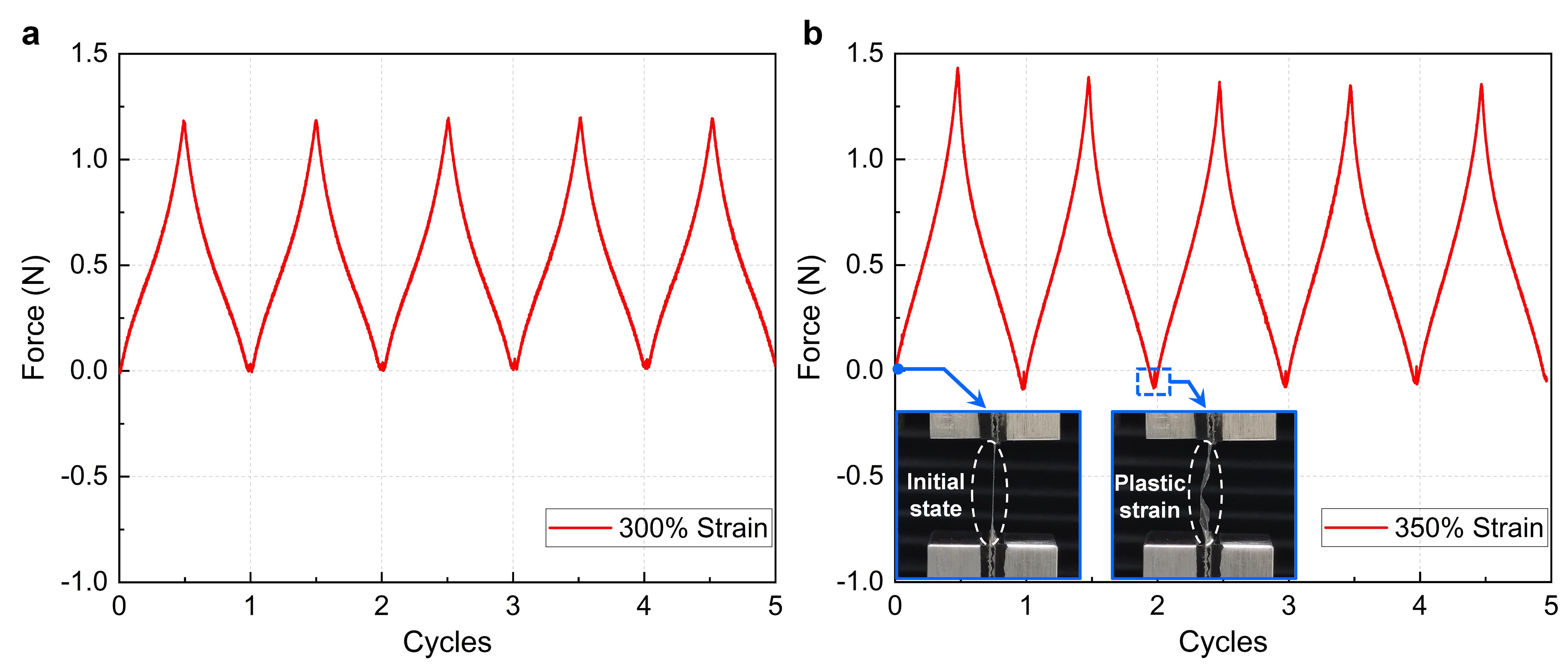


Figure S8. Mechanical response of ionic substrates under dynamic tensile strain. a) 300% strain. b) 350% strain.


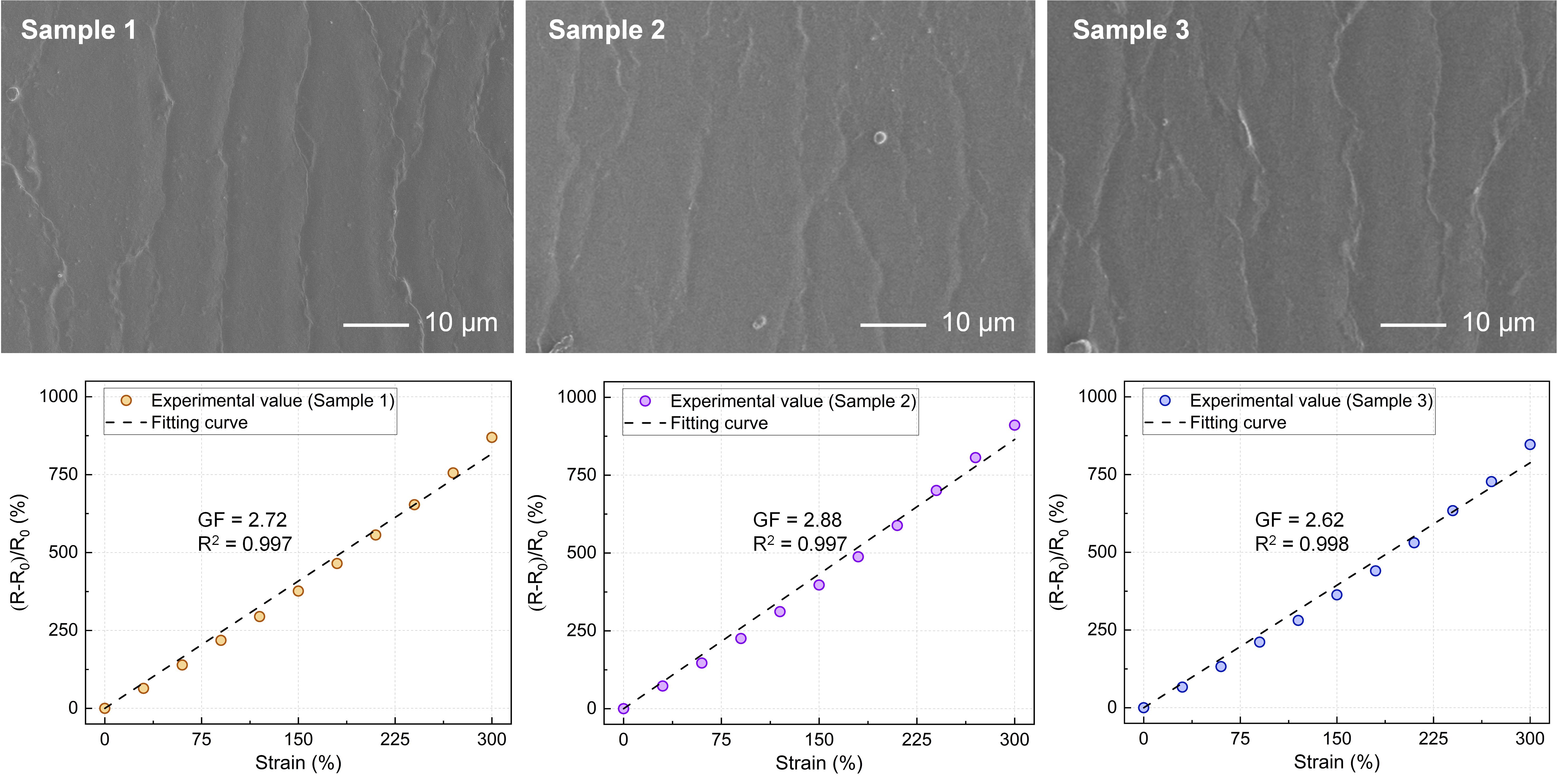


Figure S9. Surface morphology and sensing performance of different batches. Scale bar: 10 µm.


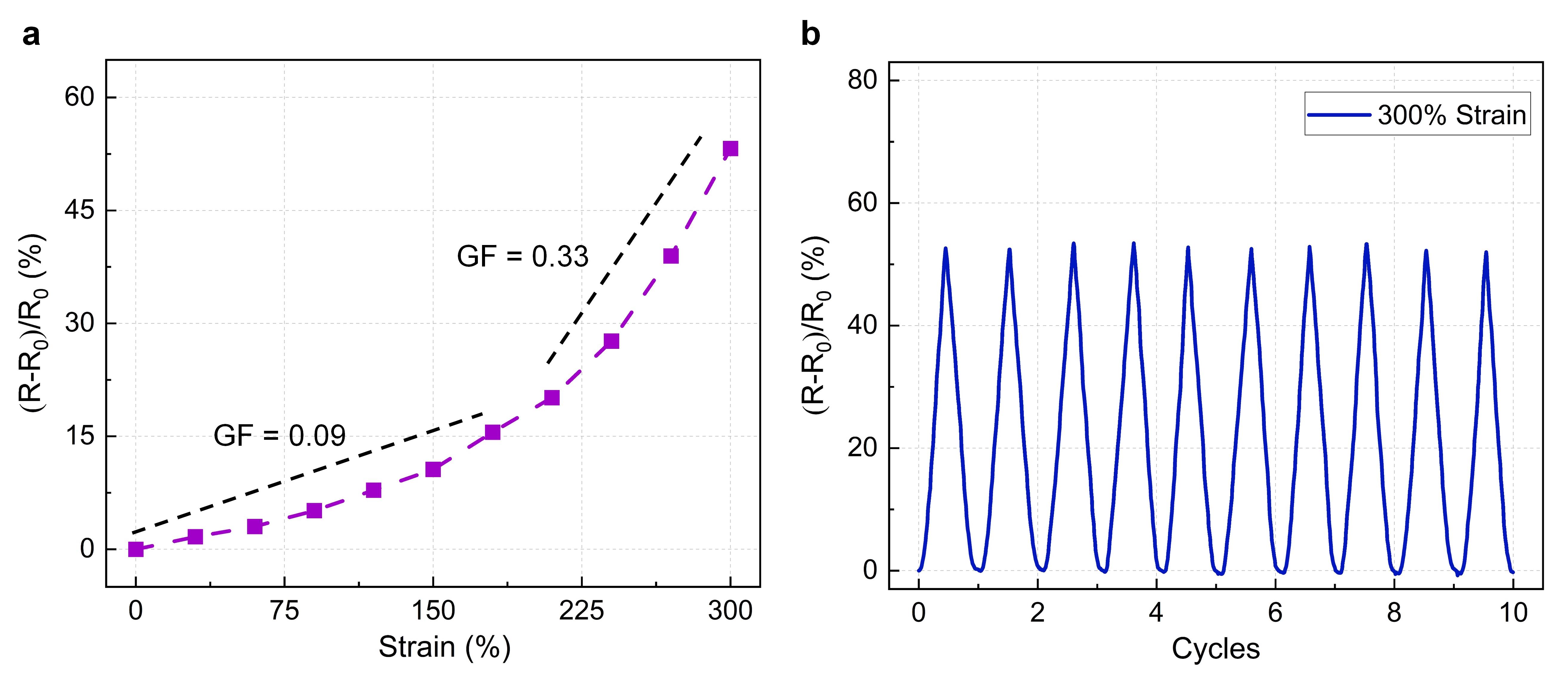


Figure S10. Electrical characterization of ionic gel. a) Resistance response of ionic gel under 300% strain. b) Dynamic resistance tests under 300% strain.


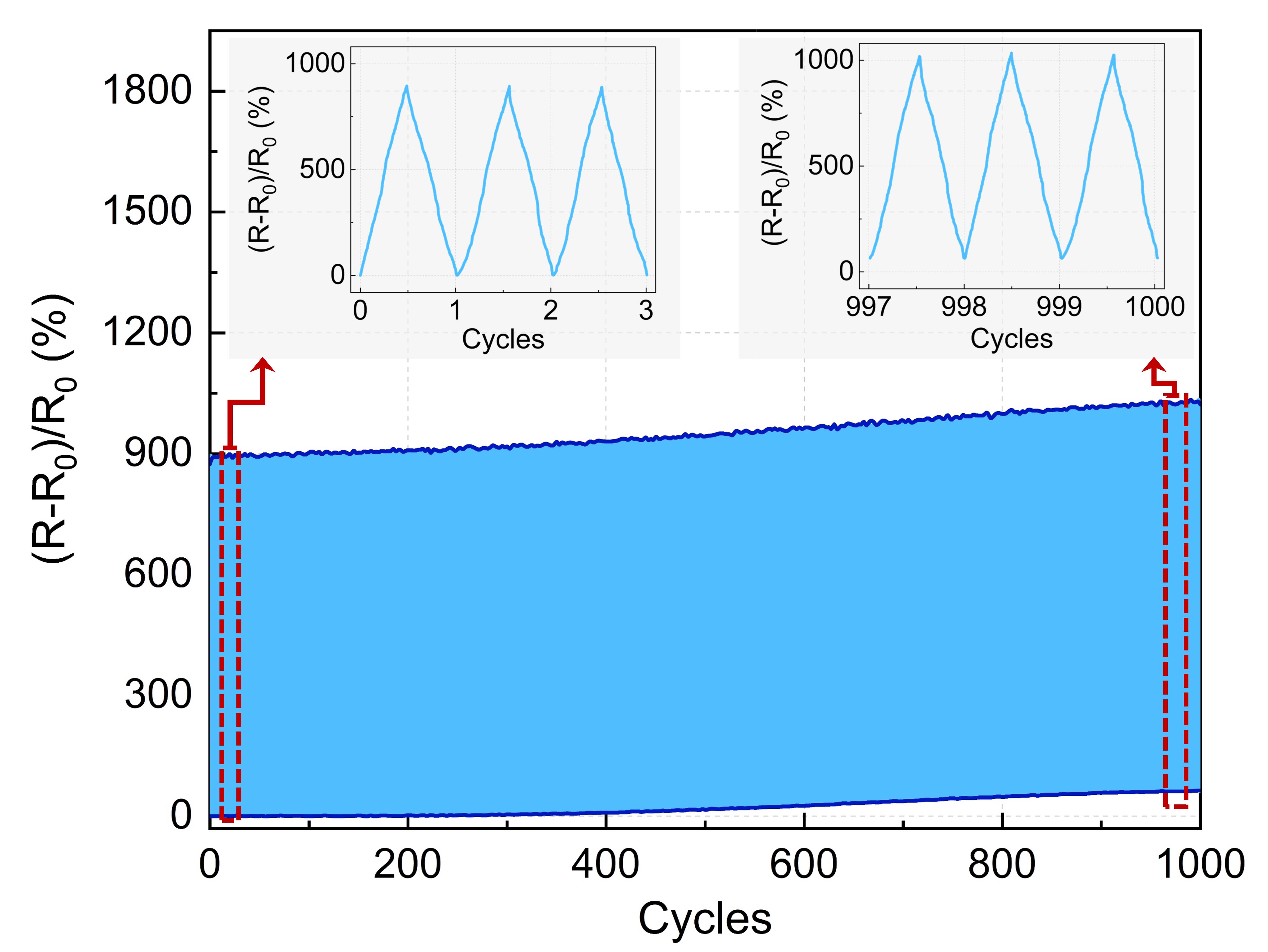


Figure S11. Durability test of i-PEDOT:PSS (300% strain).


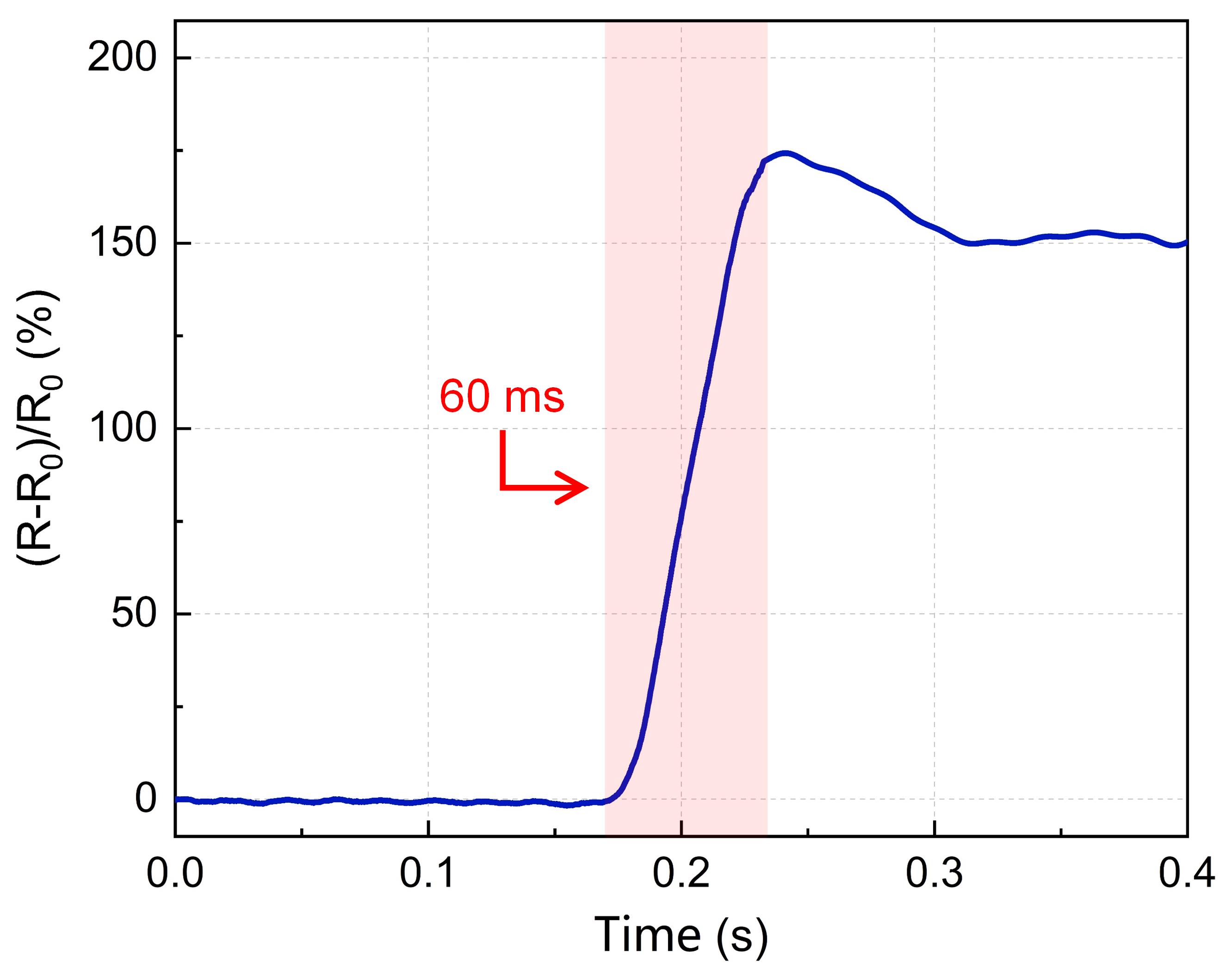


Figure S12. Transient response of i-PEDOT:PSS under 50% strain.


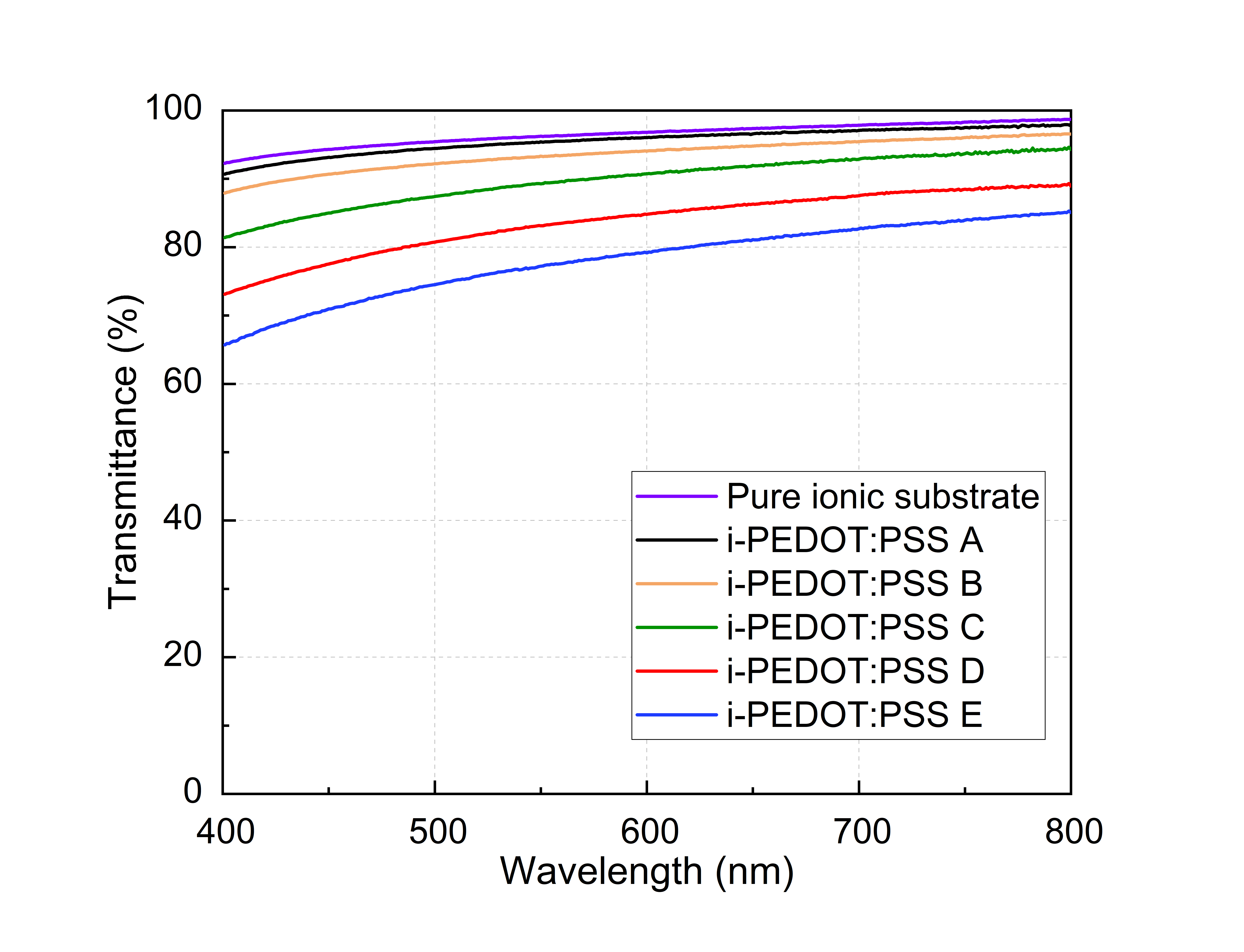


Figure S13. Optical transmittance of i-PEDOT:PSS with different PEDOT:PSS spin-coating parameters. The detailed parameters and related properties are listed in Table S1. Combining with data in Table S1, the parameters of i-PEDOT:PSS B are ultimately selected.





Figure S14. Thickness of the PEDOT:PSS layer prepared under different spin-coating parameters. a) Schematic diagram of the experimental setup. b)-f) Experimental results (detailed spin-coating parameters in Table S2).


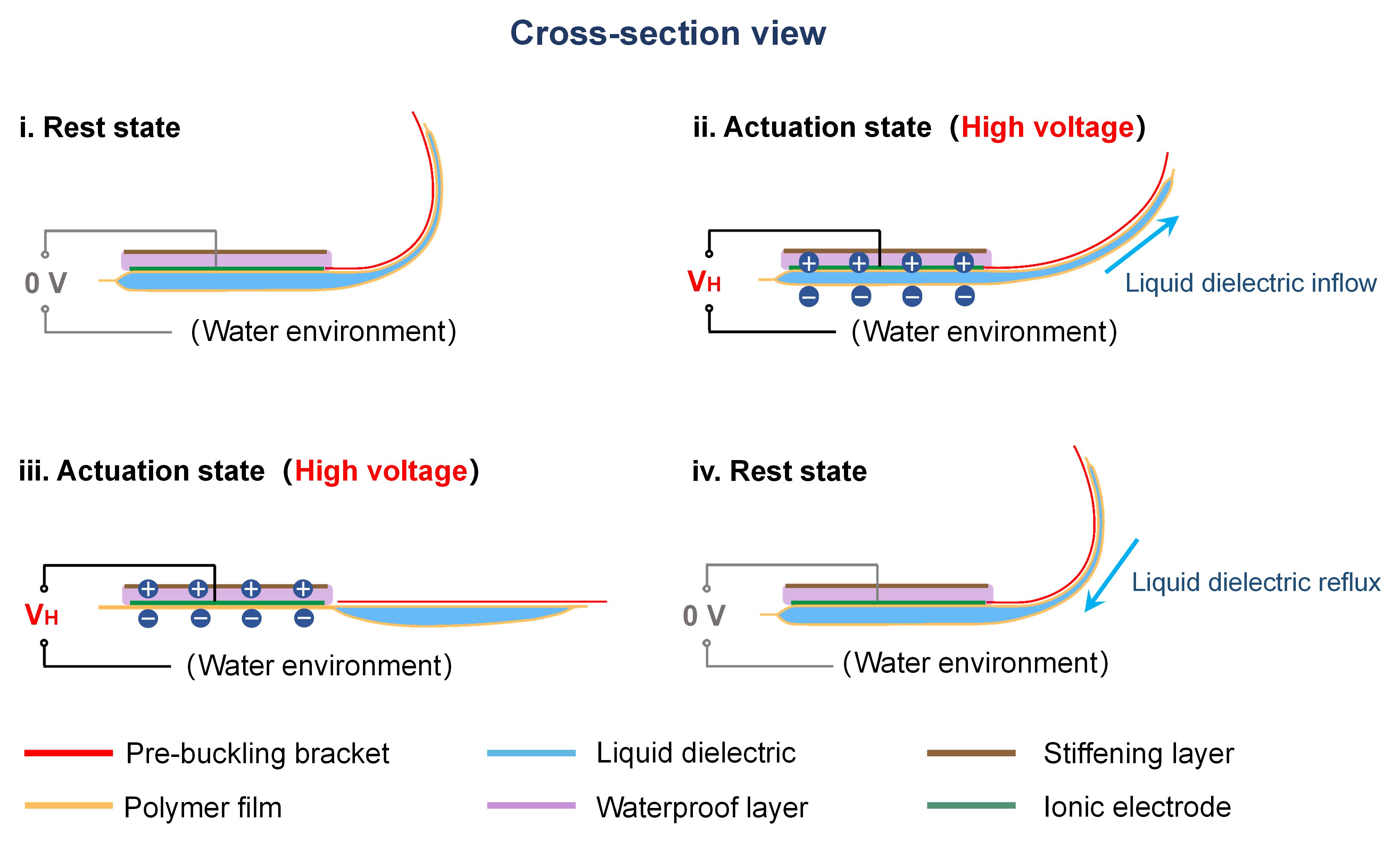


Figure S15. Actuation mechanism of the electro-hydraulic actuators.


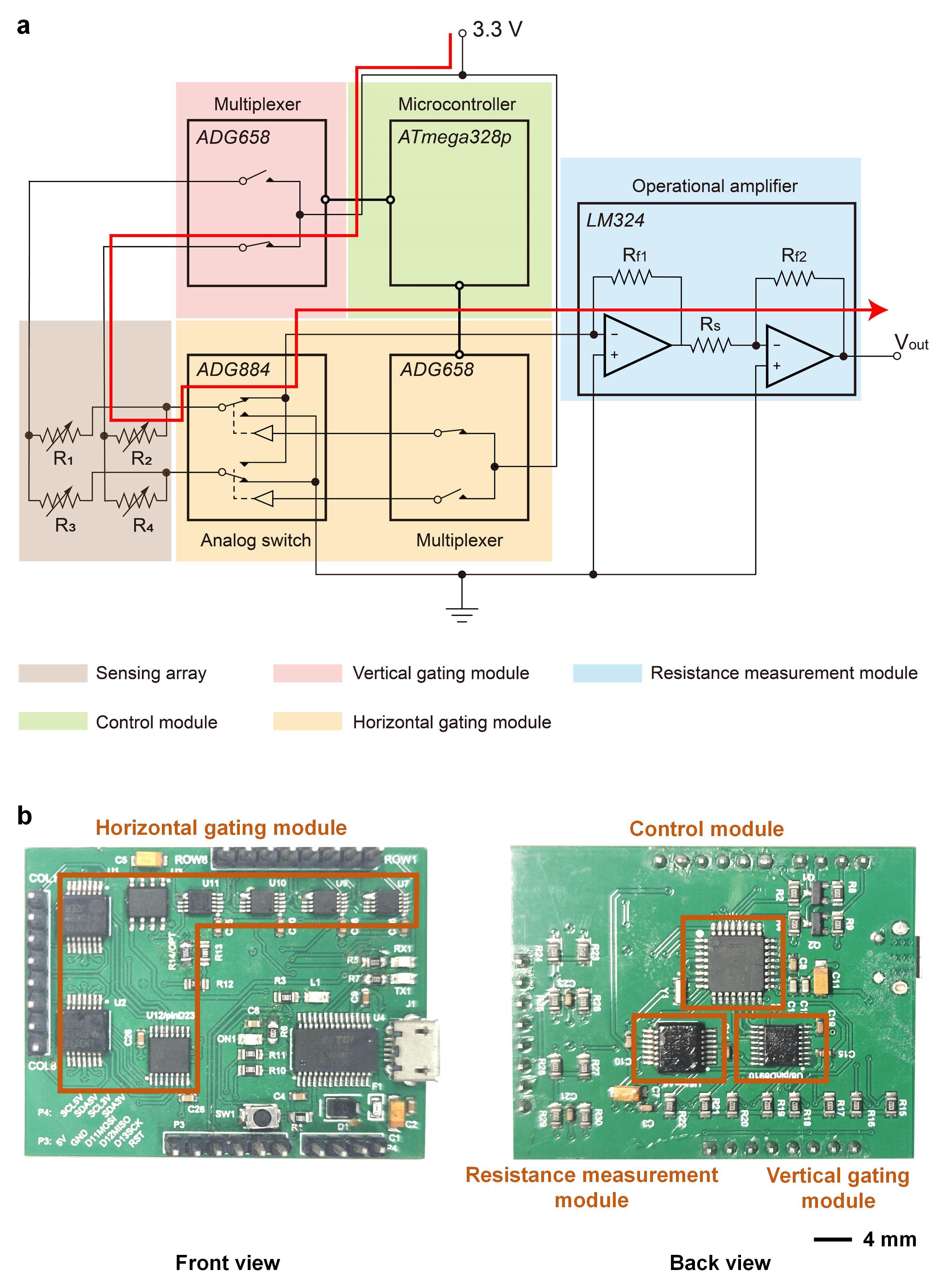


Figure S16. Multichannel strain signal acquisition circuit. a) Schematic diagram of the circuit. b) Photograph of the circuit.


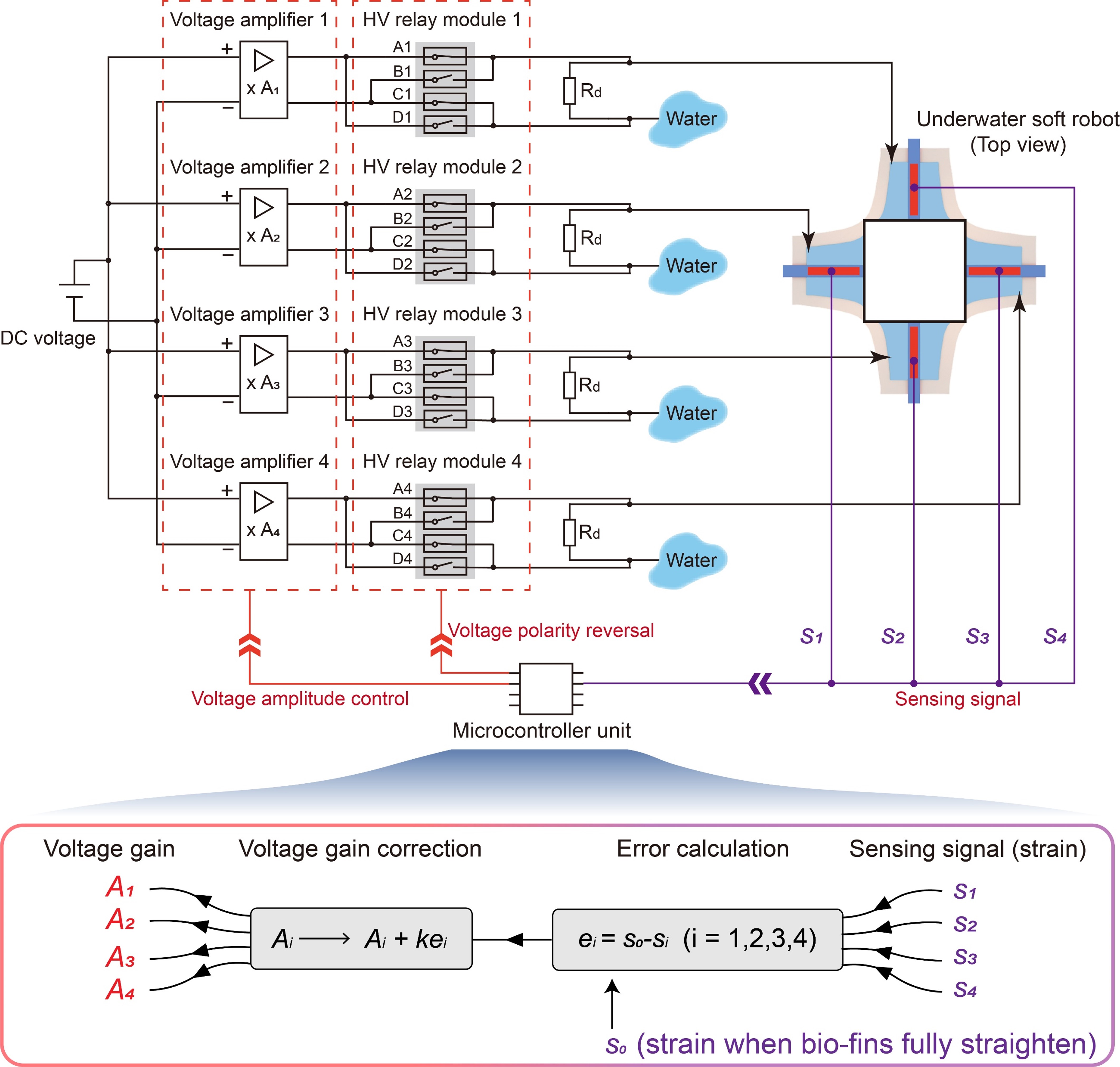


Figure S17. Negative feedback control strategy for an underwater soft robot with i-PEDOT:PSS strain sensors.


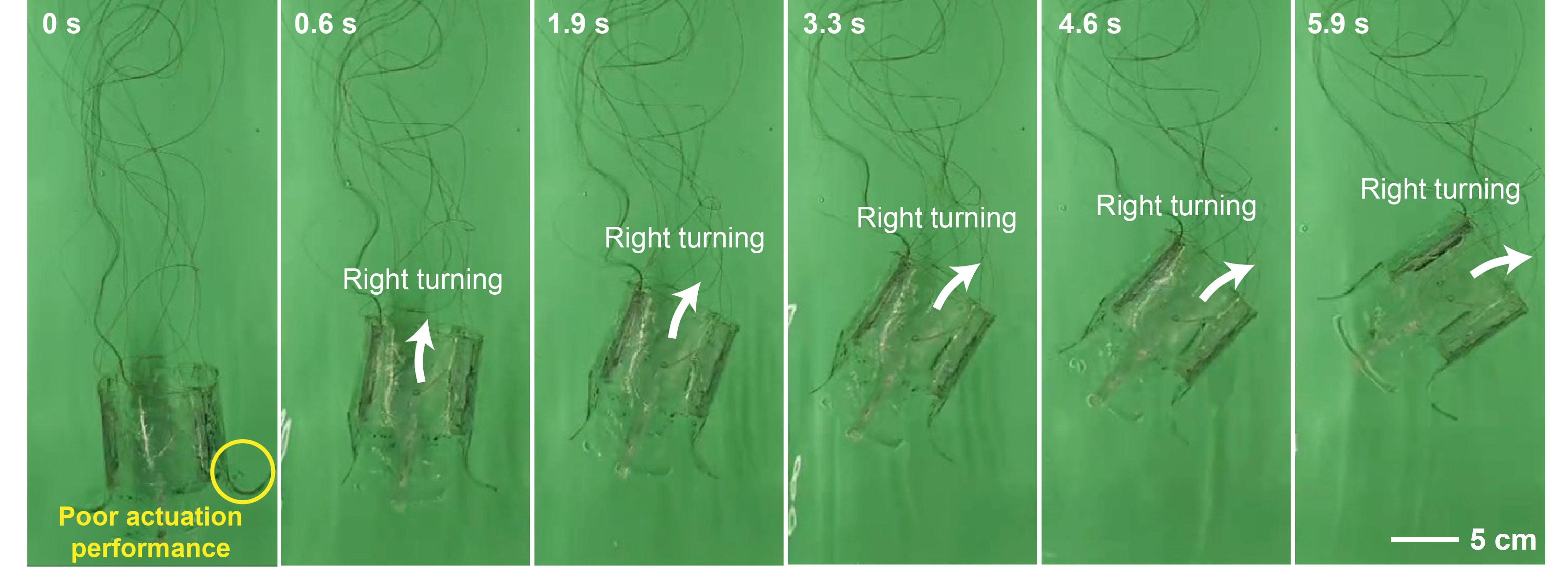


Figure S18. Trajectory deviations of an underwater soft robot without integrated strain sensors. Scale bar: 5 cm.


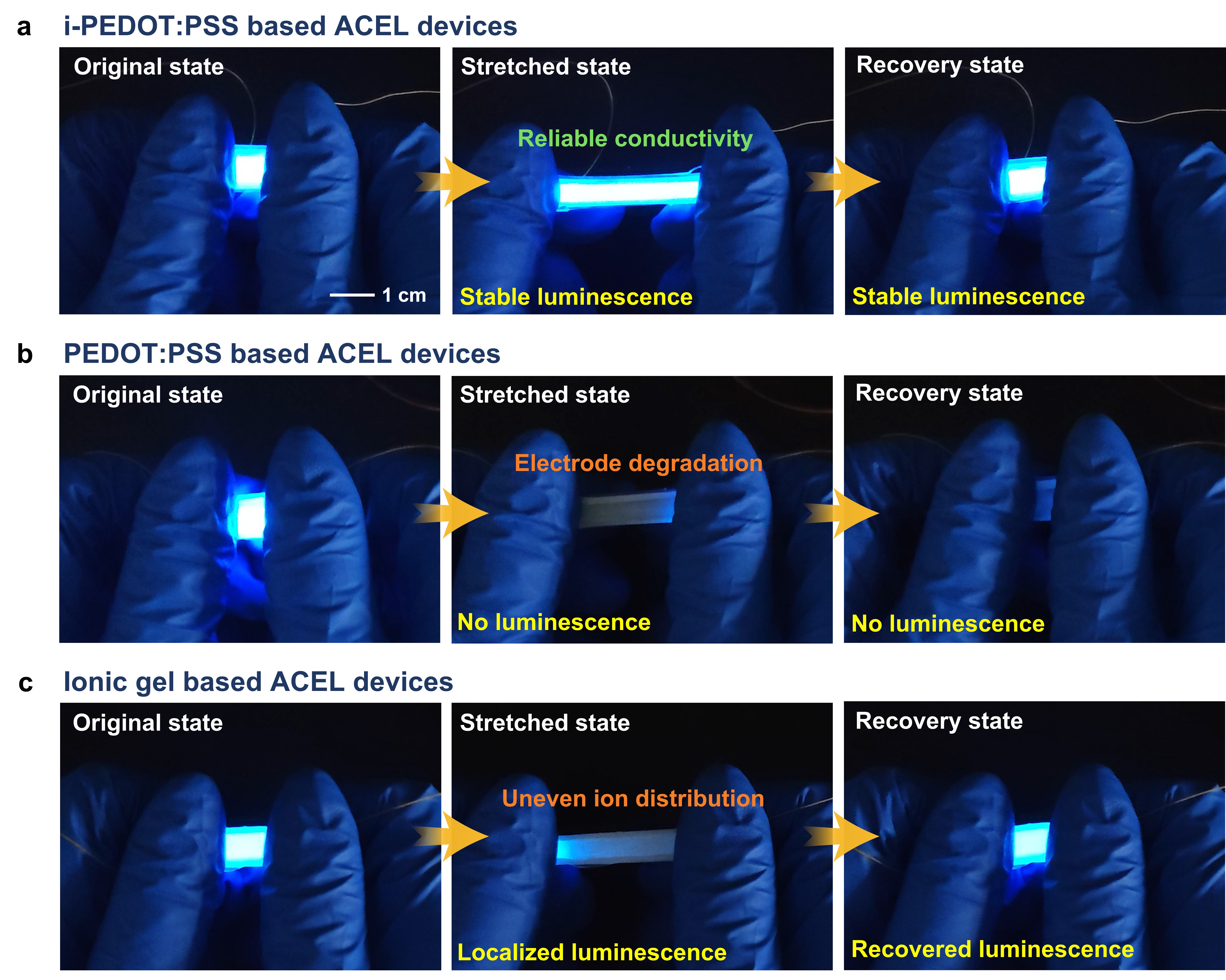


Figure S19. Luminance performance of alternating current electroluminescent (ACEL) devices based on a) i-PEDOT:PSS electrode, b) PEDOT:PSS electrode, and c) ionic gel electrode. Scale bar: 1 cm.


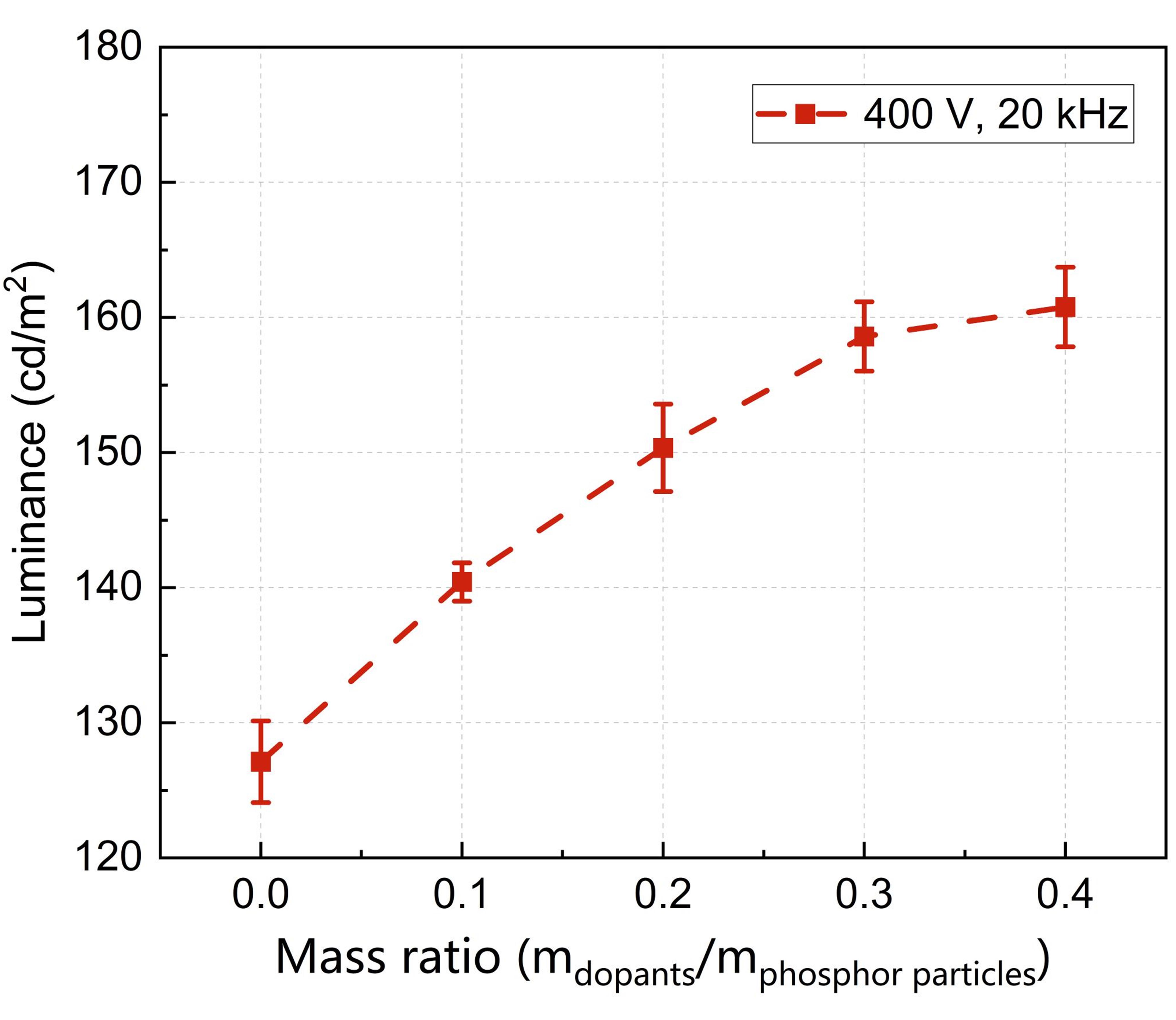


Figure S20. Correlations between luminance of ACEL devices and mass ratio in the EL layer. Data represent the mean ± s.d. (n = 3 independent samples).


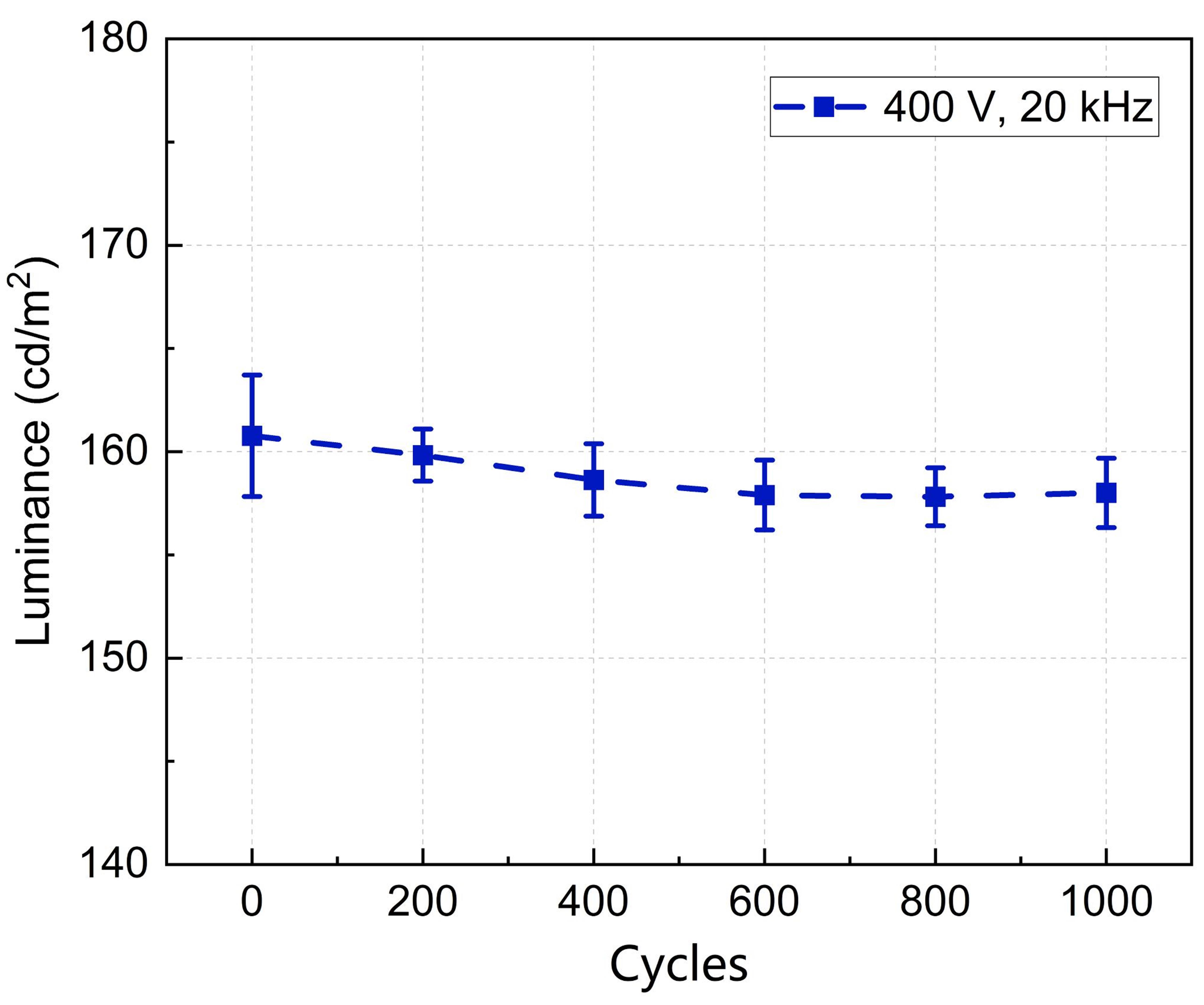


Figure S21. Durability test of the ACEL devices under dynamic strain of 50%. Data represent the mean ± s.d. (n = 3 independent samples).





Figure S22. Circuit for voltage supply to ACEL devices. a) Schematic diagram of the circuit. b) Photograph of the circuit.


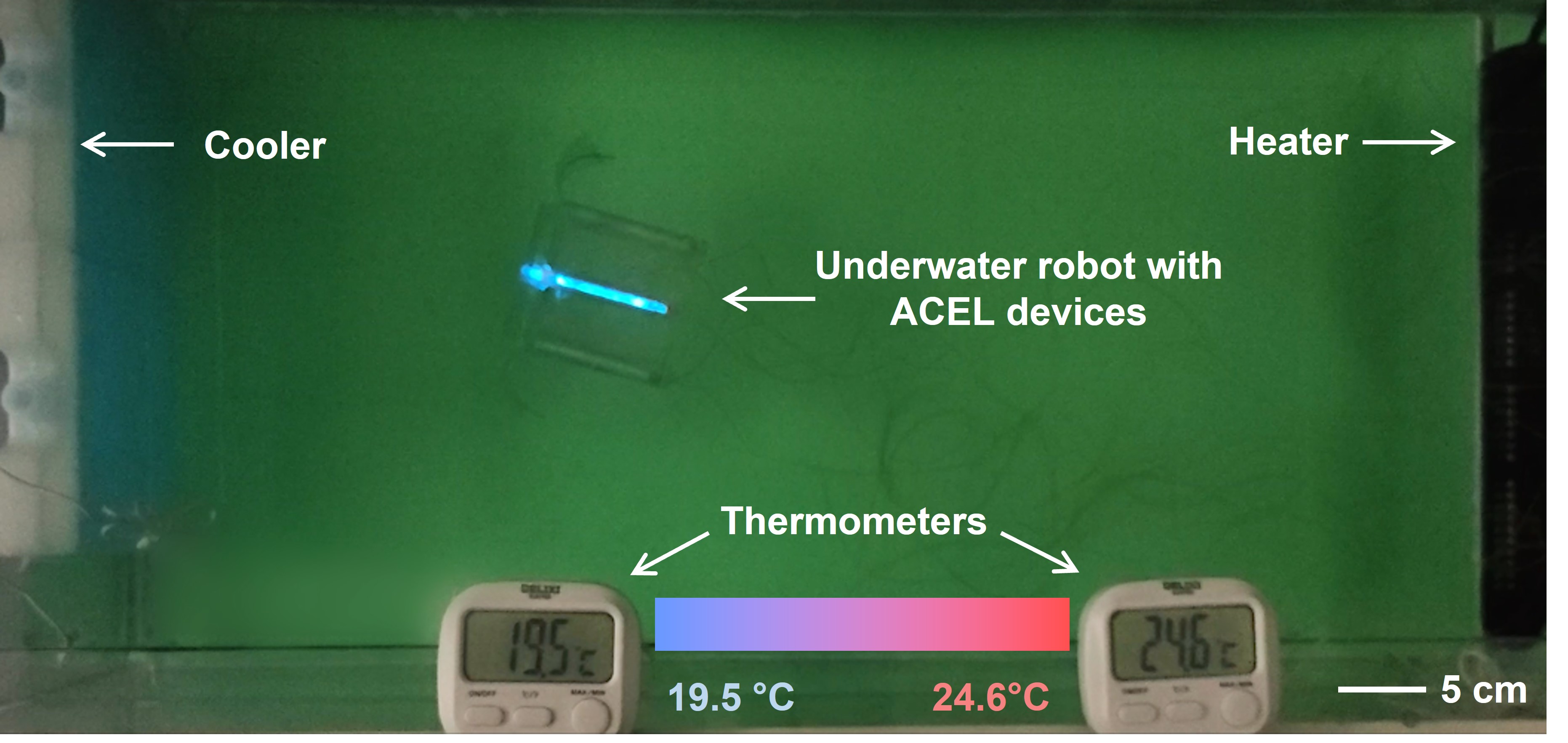


Figure S23. Experimental setup for temperature sensing and visible light communication applications. Scale bar: 5 cm.


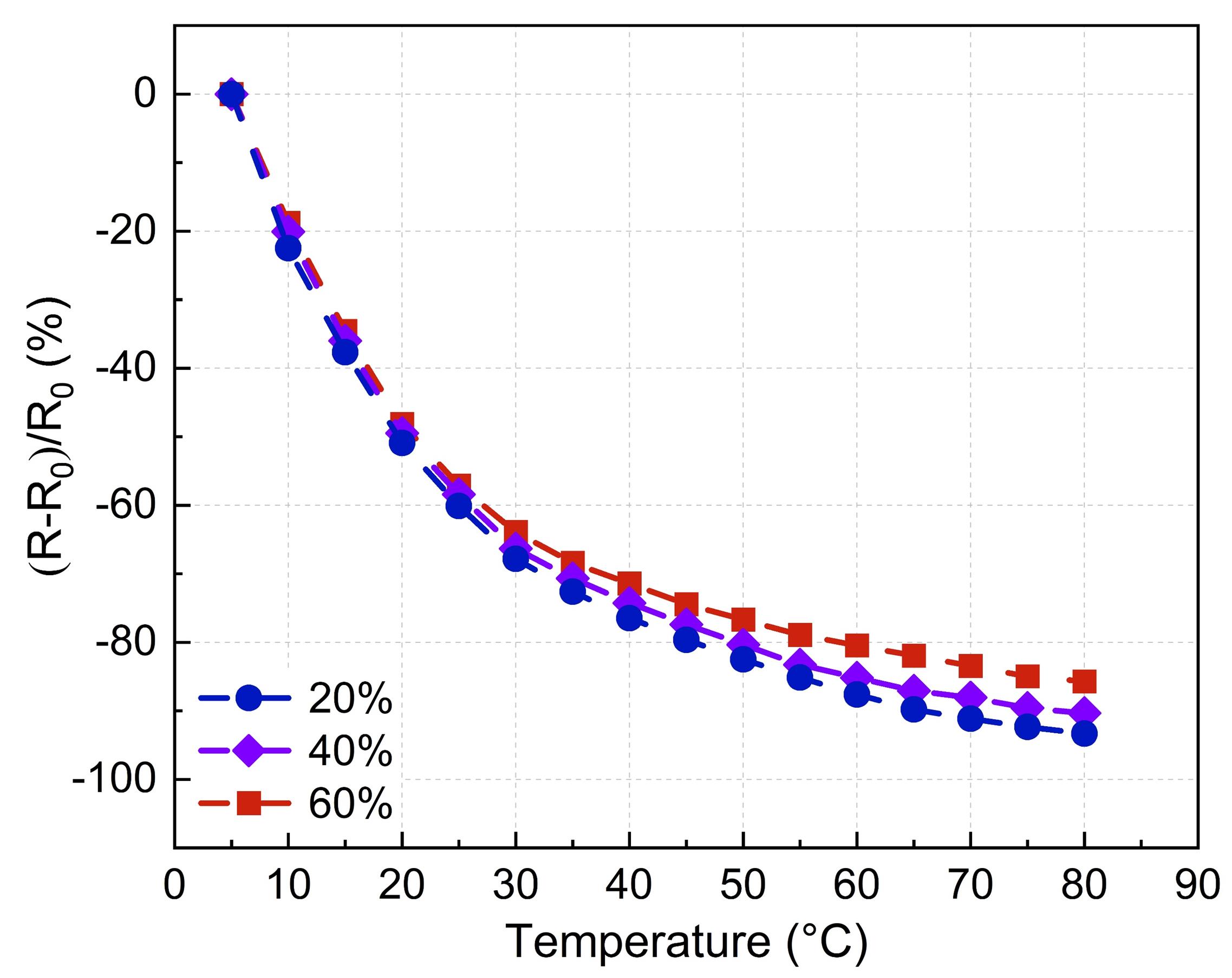


Figure S24. The resistance–temperature curves of ionic gels with different ionic liquid fractions.


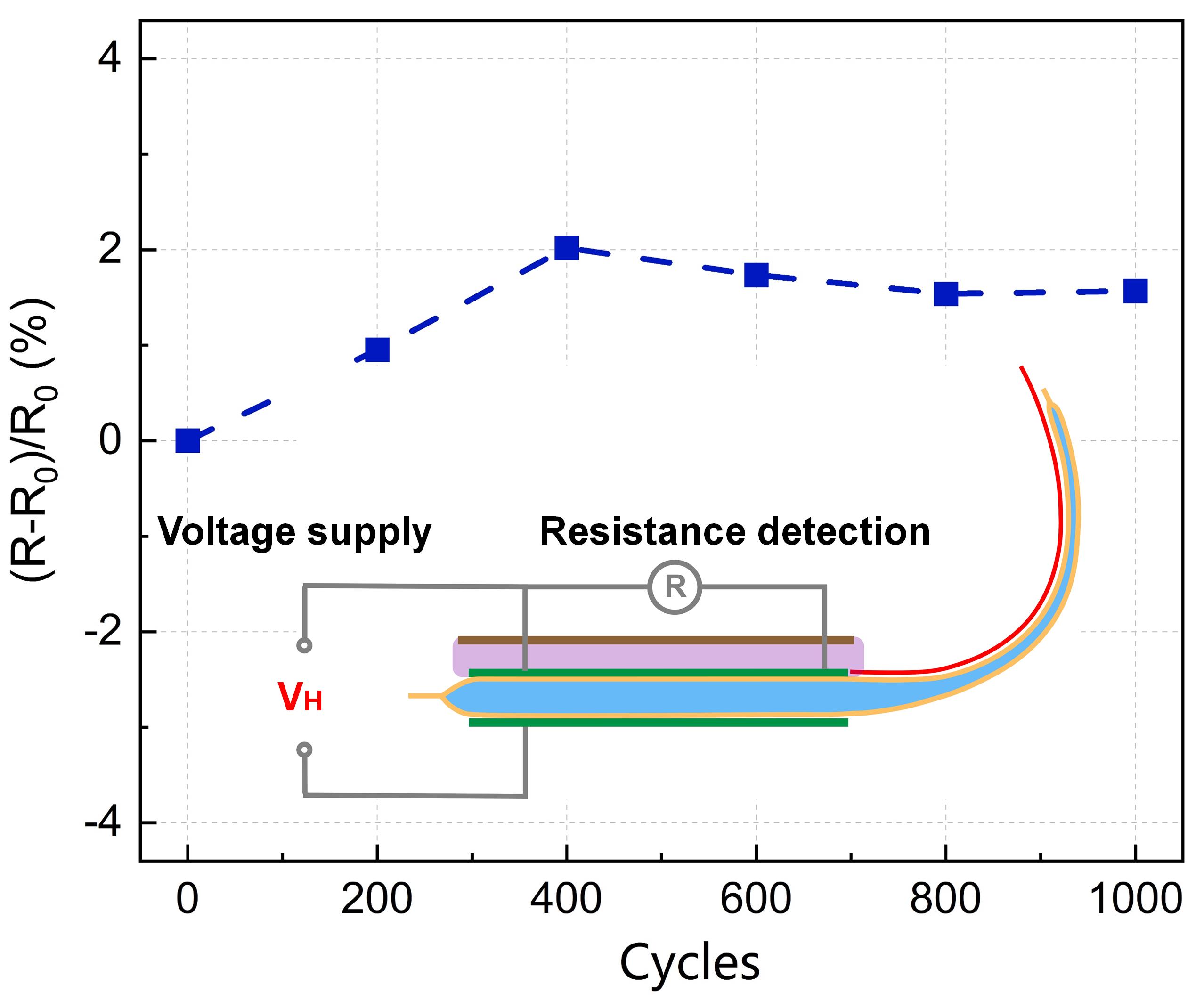


Figure S25. Resistance variation of the ionic gel during continuous high-voltage actuation.


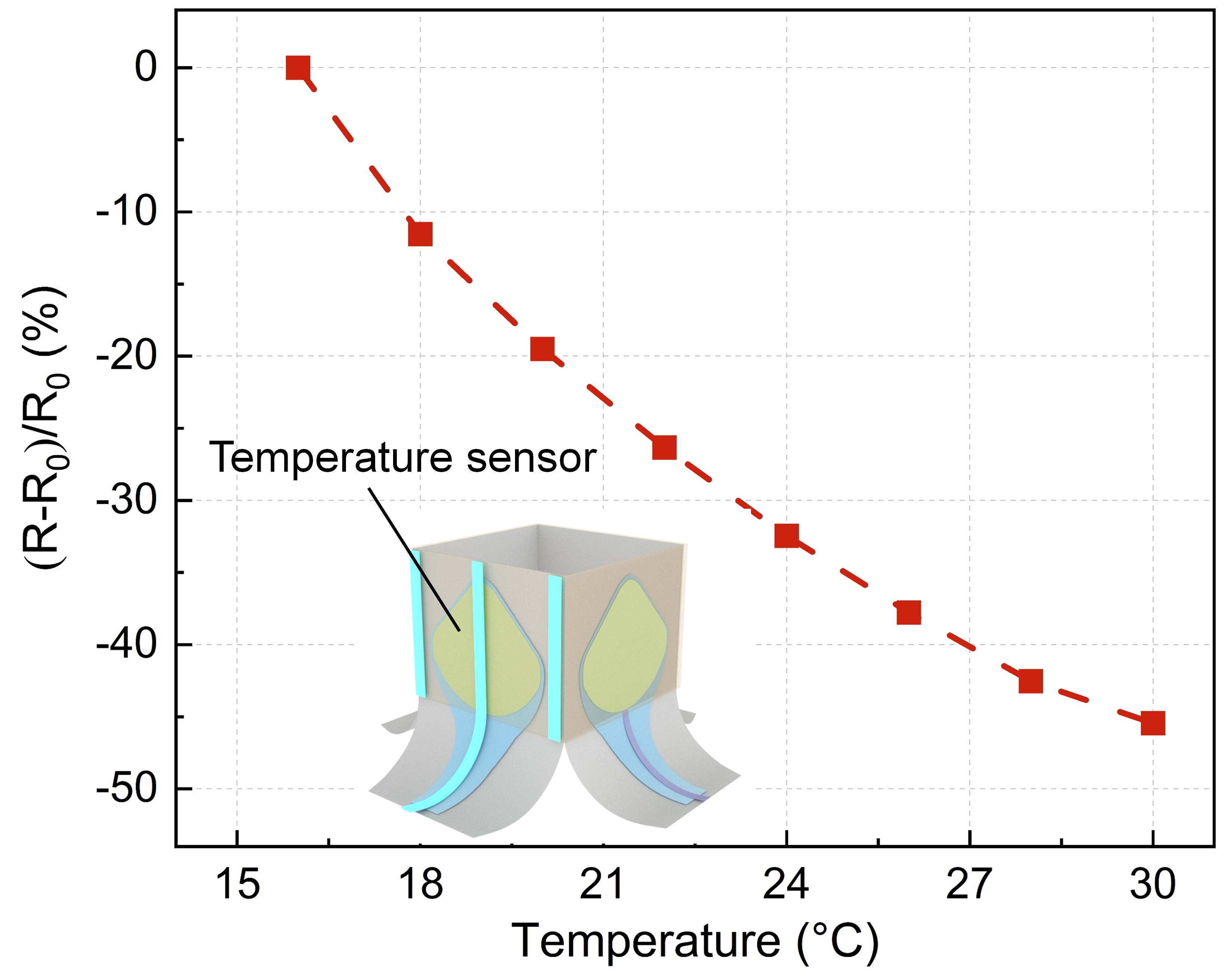


Figure S26. Resistance-temperature curve of ionic gel (20% ionic liquid fraction) on the underwater soft robot at 15–30°C.





Figure S27. Fabrication process of the EL layer in ACEL devices.

Table S1. Comparison of PEDOT:PSS film on pure TPU and ionic substrate

| **Substrate** | **Sample** | **Thickness of PEDOT:PSS (μm)** | **Sheet resistance (Ω/sq)**^a)^ |
| --- | --- | --- | --- |
| TPU | Sample 1 | 0.423 | 48 |
|  | Sample2 | 0.436 | 40 |
|  | Sample 3 | 0.409 | 61 |
| Ionic substrate | Sample 1 | 0.417 | 36 |
|  | Sample 2 | 0.435 | 28 |
|  | Sample 3 | 0.408 | 43 |

^a)^ The sheet resistance is measured without pre-stretching to avoid open-circuit failure in the TPU-based PEDOT:PSS film.

Table S2. Properties of i-PEDOT:PSS with different PEDOT:PSS spin-coating parameters

| **Sample** | **Spin-coating time (s)** | **Spin speed (rpm)^a)^** | **Thickness of PEDOT:PSS**  **(μm)** | **Transmittance (%)^b)^** | **Conductivity (S/cm)** |
| --- | --- | --- | --- | --- | --- |
| Ionic substrate | - | - | - | 96 | 0.0001 |
| i-PEDOT:PSS A | 6 | 1000 | 0.14 | 95 | <27.34 ^c)^ |
| **i-PEDOT:PSS B** | **4** | **1000** | **0.42** | **93** | **31.25** |
| i-PEDOT:PSS C | 4 | 800 | 0.54 | 89 | 28.02 |
| i-PEDOT:PSS D | 4 | 500 | 2.37 | 83 | 7.19 |
| i-PEDOT:PSS E | 3 | 500 | 3.32 | 77 | 6.01 |

^a)^ Revolutions per minute (rpm)

^b)^ Transmittance at 550 nm

^c)^ Significant variation in conductivity is observed across the film, indicating poor uniformity

Table S3. Comparison with previously reported stretchable transparent films for strain-sensing application

| **Materials** | **Linear sensing range (%)** | **Transmittance**  **(%)^a)^** | **Gauge factor** | **Elongation**  **(%)** | **Durability**  **(cycles)** |
| --- | --- | --- | --- | --- | --- |
| Ionic gel^[1]^ | 50 | 90 | 1.95 | 1200 | 500 |
| Ionic gel^[2]^ | 100 | 90 | 1.49 | 1033 | 150 |
| Ag-PDMS^[3]^ | 15 | 79 | 14.3 | 35 | 800 |
| Hydrogel^[4]^ | 80 | 92 | 7.05 | 233 | 87 |
| Graphene-PDMS^[5]^ | 10 | 48 | 70 | 20 | 4000 |
| AgNWs-PU^[6]^ | 25 | 90 | 11.2 | 500 | 5816 |
| Ionic liquid-PDMS^[7]^ | 65 | 90.5 | 0.096 | 80 | 10 |
| PEDOT:PSS-AgNWs-PDMS^[8]^ | 20 | 85 | 10.2 | 100 | 2000 |
| **PEDOT:PSS-Ionic gel ^b)^** | **300** | **93** | **2.87** | **1360** | **5000** |

^a)^ Transmittance at 550 nm

^b)^ This work

Table S4. Luminance states of ACEL devices in the application of visual light communication

| **Luminance states of ACEL devices** | | | **Meaning** |
| --- | --- | --- | --- |
| Central | Left | Right |  |
| On | On | Off | Signal output (binary “0”) |
| On | Off | On | Signal output (binary “1”) |
| On | Off | Off | Temperature detection |
| Off | Off | Off | Robot idle |

**Movie S1. Stretchability of i-PEDOT:PSS film**

The i-PEDOT:PSS inherits the stretchability of ionic gel, with fracture occurring at a strain of approximately 1360%.

**Movie S2. The i-PEDOT:PSS-based strain sensing for soft robotic posture correction**

Despite being programmed for linear motion, the robot deviated rightward as a result of diminished performance in bio-fin C, which was promptly detected by the i-PEDOT:PSS sensors. Based on the strain-sensing signals from the four channels, the negative feedback control strategy was then implemented by increasing the driving voltage of bio-fin C from 6 kV to 6.8 kV. This adjustment balanced the performance of the four actuators and achieved linear motion of the underwater soft robot. In contrast, underwater soft robots without strain sensing may lead to trajectory deviations.

**Movie S3. Comparative luminance performance of ACEL devices employing various electrodes**

The i-PEDOT:PSS-based ACEL devices exhibit stable luminance performance under stretching, twisting, and bending, whereas ACEL devices based on ionic gel electrodes and pristine PEDOT:PSS fail to emit light evenly when stretched.

**Movie S4. The i-PEDOT:PSS-based ACEL devices for robot motion tracking**

Three ACEL devices were mounted on the robot: a longer middle device, operated at 200 V for continuous illumination to facilitate real-time localization, and two shorter side ACEL devices, operated at 400 V for higher luminance and used for signaling left or right turns via flashing. By designating the top of the middle ACEL device as a tracking marker, the robot's position and trajectory could be dynamically captured.

**Movie S5. The i-PEDOT:PSS-based ACEL devices for visible light communication**

A temperature gradient was established within a water tank by positioning a heater and a cooler at opposite ends, resulting in measured temperatures of 19.5°C and 24.6°C at the starting and ending points of robot’s motion trajectory respectively. The robot employed ion gel with intrinsic temperature sensitivity. During locomotion, the robot performed temperature measurements after each propulsion, indicated by the illumination of the central ACEL device. The two side ACEL devices encoded temperature information at the start and end positions using binary signals—illumination of the respective device representing binary '1' or '0'. The measurement error is within 7% relative to the standard thermometer.

**SI References**

[1] Y. Yuan, J. Zhou, G. Lu, J. Sun, L. Tang, *ACS Appl. Polym. Mater.* **2021**, *3*, 1610.

[2] J. Lan, B. Zhou, C. Yin, L. Weng, W. Ni, L.-Y. Shi, *Polymer* **2021**, *231*.

[3] W. Zhou, Y. Li, P. Li, J. Chen, R. Xu, S. Yao, Z. Cui, R. Booth, B. Mi, D. Wang, Y. Ma, W. Huang, *Adv. Mater. Technol.* **2019**, *4*.

[4] L. Zhao, Q. Ling, X. Fan, H. Gu, *ACS Appl. Mater. Interfaces* **2023**, *15*, 40975.

[5] X. Liu, D. Liu, J. H. Lee, Q. Zheng, X. Du, X. Zhang, H. Xu, Z. Wang, Y. Wu, X. Shen, J. Cui, Y. W. Mai, J. K. Kim, *ACS Appl. Mater. Interfaces* **2019**, *11*, 2282.

[6] Y. X. Song, W. M. Xu, M. Z. Rong, M. Q. Zhang, *J. Mater. Chem. A* **2019**, *7*, 2315.

[7] N. Jiang, D. Hu, Y. Xu, J. Chen, X. Chang, Y. Zhu, Y. Li, Z. Guo, *Adv. Compos. Hybrid Mater.* **2021**, *4*, 574.

[8] G. Shen, B. Chen, T. Liang, Z. Liu, S. Zhao, J. Liu, C. Zhang, W. Yang, Y. Wang, X. He, *Adv. Electron. Mater.* **2020**, *6*.
